# Anion-dependent ion-pairing assemblies of triazatriangulenium cation that interferes with stacking structures

**DOI:** 10.3762/bjoc.20.215

**Published:** 2024-10-10

**Authors:** Yohei Haketa, Takuma Matsuda, Hiromitsu Maeda

**Affiliations:** 1 Department of Applied Chemistry, College of Life Sciences, Ritsumeikan University, Kusatsu 525–8577, Japan

**Keywords:** charged π-electronic systems, ion pairs, single-crystal X-ray analysis, solid-state assemblies, triazatriangulenium cation

## Abstract

Ion pairs of *N*-(2,6-dimethylphenyl)-substituted triazatriangulenium (TATA^+^) cation with various counteranions were synthesized to investigate the interactions for the bulky cation. Single-crystal X-ray analysis of the TATA^+^ ion pairs revealed solid-state ion-pairing assemblies without stacking at the cationic π-planes. The TATA^+^ cation showed counteranion-dependent assembly structures, with smaller counteranions located at the top of TATA^+^ and bulkier counteranions displaced from the TATA^+^ plane to interact with the surrounding TATA^+^.

## Introduction

**Figure 1:**
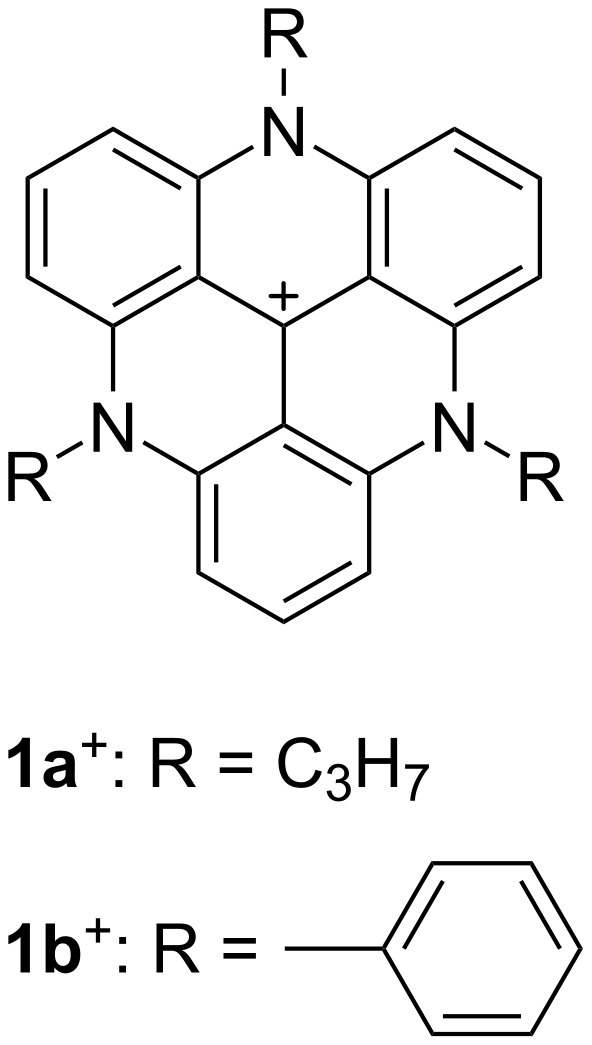
Triazatriangulenium cations **1a**^+^ and **1b**^+^.

Triangulenium cations [1–2] have been widely investigated in the past decades for application as fluorescent dyes [3–5], chirality inducers [6–8], acceptors for photoinduced electron transfer (PET) [9–10], and components of supramolecular assemblies [11–14]. Owing to the stable π-electronic systems showing unique electronic and electrooptical properties, various derivatives have been synthesized via peripheral modifications of the triangulenium cations. In particular, various functional units can be introduced at the N-positions of azatriangulenium cations. For example, *N*-alkyl-substituted triazatriangulenium (TATA^+^) cations, used as visible light fluorescent dyes, have been synthesized via a nucleophilic aromatic substitution (S_N_Ar) reaction with primary alkylamines (e.g., **1a**^+^; Figure 1) [15–16]. The highly planar geometry of the TATA^+^ core unit induces π–π stacking structures in single-crystal and film states, as seen in the BF_4_^–^ ion pairs of *N*-alkyl-substituted TATA^+^ cations.

The combination of TATA^+^ cations with counteranions afforded various ion pairs, resulting in characteristic assembled structures [17]. The ion pair comprising *N*-propyl-substituted **1a**^+^ and pentacyanocyclopentadienide (PCCp^−^) [18–19] formed a charge-by-charge assembled structure, with alternate stacking of TATA^+^ and PCCp^−^ through *^i^*π–*^i^*π interactions in the crystal state [20–21]. Charge-by-charge stacking assemblies of **1a**^+^ and porphyrin-based π-electronic anions showed the crystal-state PET between proximally located anions and cations [21]. Moreover, ion-pairing assemblies of **1a**^+^ and the π-electronic receptor–anion complexes as pseudo-π-electronic anions exhibited electric conductive properties [22–25]. The structures and electronic states of ions directly affect the arrangement of constituent ions in the assemblies and resulting properties. Thus, the design and synthesis of ion pairs are important for preparing functional ion-pairing assemblies.

In contrast to *N*-alkyl-substituted TATA^+^ cations, only a few *N*-aryl-substituted TATA^+^ cations have been reported to date [26–29]. This is probably because the introduction of *N*-aryl units during the synthesis is rendered difficult owing to steric hindrances. For example, *N*-phenyl-substituted TATA^+^ cation **1b**^+^ (Figure 1) [26] exhibited slightly red-shifted absorption and emission, with a higher quantum efficiency compared to those of *N*-alkyl-substituted TATA^+^ cations. Moreover, the *N*-aryl-substituted TATA^+^ cations have been used as the building units of porous organic polymers for capturing CO_2_ [27]. Cations with characteristic electron-deficient planar triangular geometries were used as scaffolds for various supramolecular cage structures [28–29]. Although modifications of the *N*-aryl units as sterically hindered substituents significantly affect the assembling behaviors, their solid-state packing structures are largely unknown. In this study, ion pairs (salts) of an *N*-aryl-substituted TATA^+^ as a bulky cation with various counteranions were prepared for investigating the ion-pairing assemblies.

## Results and Discussion

**Figure 2:**
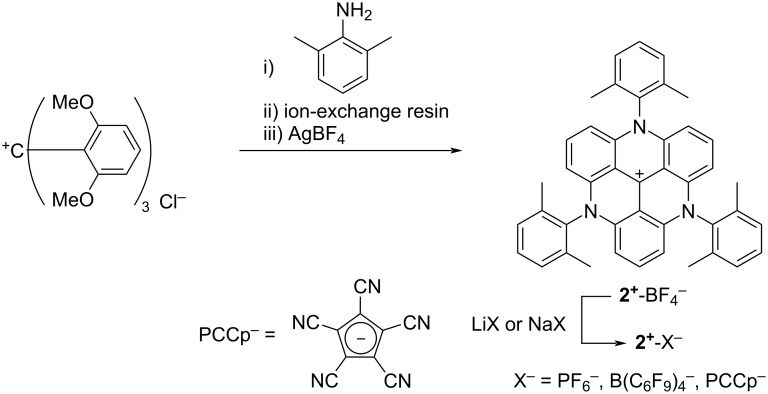
Synthesis of triazatriangulenium ion pairs **2**^+^-X^−^ (X^−^ = BF_4_^−^, PF_6_^−^, B(C_6_F_5_)_4_^−^, and PCCp^−^).

The *N*-(2,6-dimethylphenyl)-substituted triazatriangulenium cation **2**^+^ was synthesized as a BF_4_^−^ ion pair **2**^+^-BF_4_^−^ in 19% yield using a modified synthetic procedure for **2**^+^-Cl^−^ [30] (Figure 2). It should be noticed that the S_N_Ar reaction for ring closure with aniline requires a base as reported by Laursen et al. [26], whereas 2,6-dimethylaniline in this study was reacted without the use of a base. This can be explained by the high nucleophilicity of 2,6-dimethylaniline, though it is sterically hindered. As counteranions affect ion-pairing assemblies, **2**^+^-BF_4_^−^ was further treated with NaPF_6_, LiB(C_6_F_5_)_4_, and NaPCCp for the ion-pair metathesis to afford ion pairs **2**^+^-X^−^ (X^−^ = PF_6_^−^, B(C_6_F_5_)_4_^−^, and PCCp^−^) in 44–68% yields. The obtained ion pairs were characterized using ^1^H, ^13^C, and ^19^F nuclear magnetic resonance (NMR) and matrix-assisted laser desorption ionization time-of-flight mass spectrometry (MALDI–TOF MS). The synthesized ion pairs showed similar electronic properties in solution state. In CH_3_CN, **2**^+^-BF_4_^−^ exhibited UV–vis absorptions at 273, 350, and 524 nm and fluorescence emission at 607 nm upon excitation at 524 nm. The absorption band at 524 nm was mainly derived from HOMO−1/HOMO-to-LUMO transition (HOMO: highest occupied molecular orbital, LUMO: lowest unoccupied molecular orbital), as revealed by the time-dependent density functional theory (TDDFT) calculation of the optimized structure of **2**^+^ at the B3LYP/6-31+G(d,p) level of theory (Figures S22–25 in Supporting Information File 1) [31]. The absorption band at 524 nm was blue-shifted by 4 nm compared to that of *N*-phenyl-substituted **1b**^+^-BF_4_^−^ [15]. This can be ascribed to the lesser conjugation of the core TATA^+^ unit with the introduced 2,6-dimethylphenyl moieties owing to the more orthogonal arrangement in comparison with the phenyl moieties in **1b**^+^.

**Figure 3:**
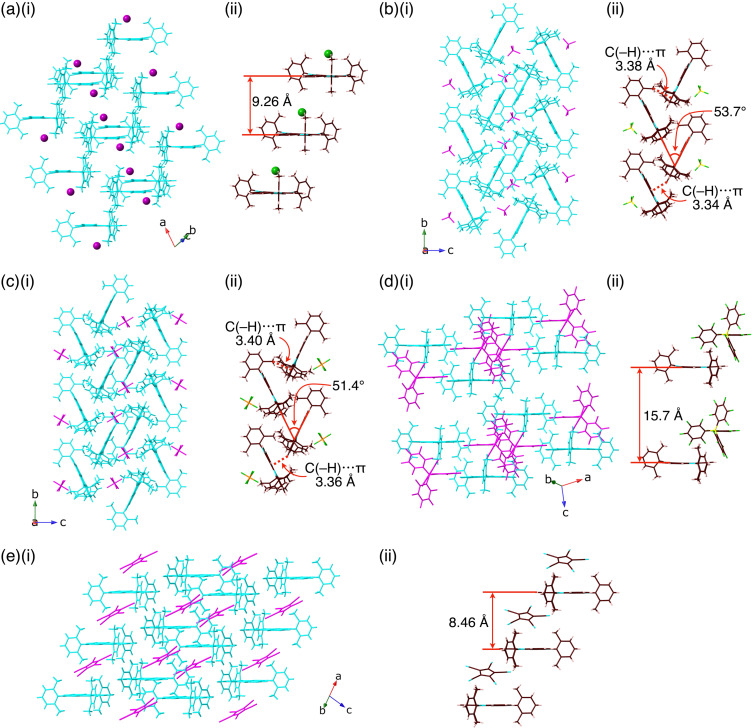
Single-crystal X-ray structures of (a) **2**^+^-Cl^−^, (b) **2**^+^-BF_4_^−^, (c) **2**^+^-PF_6_^−^, (d) **2**^+^-B(C_6_F_5_)_4_^−^, and (e) **2**^+^-PCCp^−^ as (i) packing structures and (ii) enlarged views for the columnar structures. In (i), cation and anion are represented in cyan and magenta colors, respectively. In (ii), brown, pink, yellow, blue, yellow green, orange, and green (spherical) refer to carbon, hydrogen, boron, nitrogen, fluorine, phosphorus, and chlorine, respectively.

**Figure 4:**
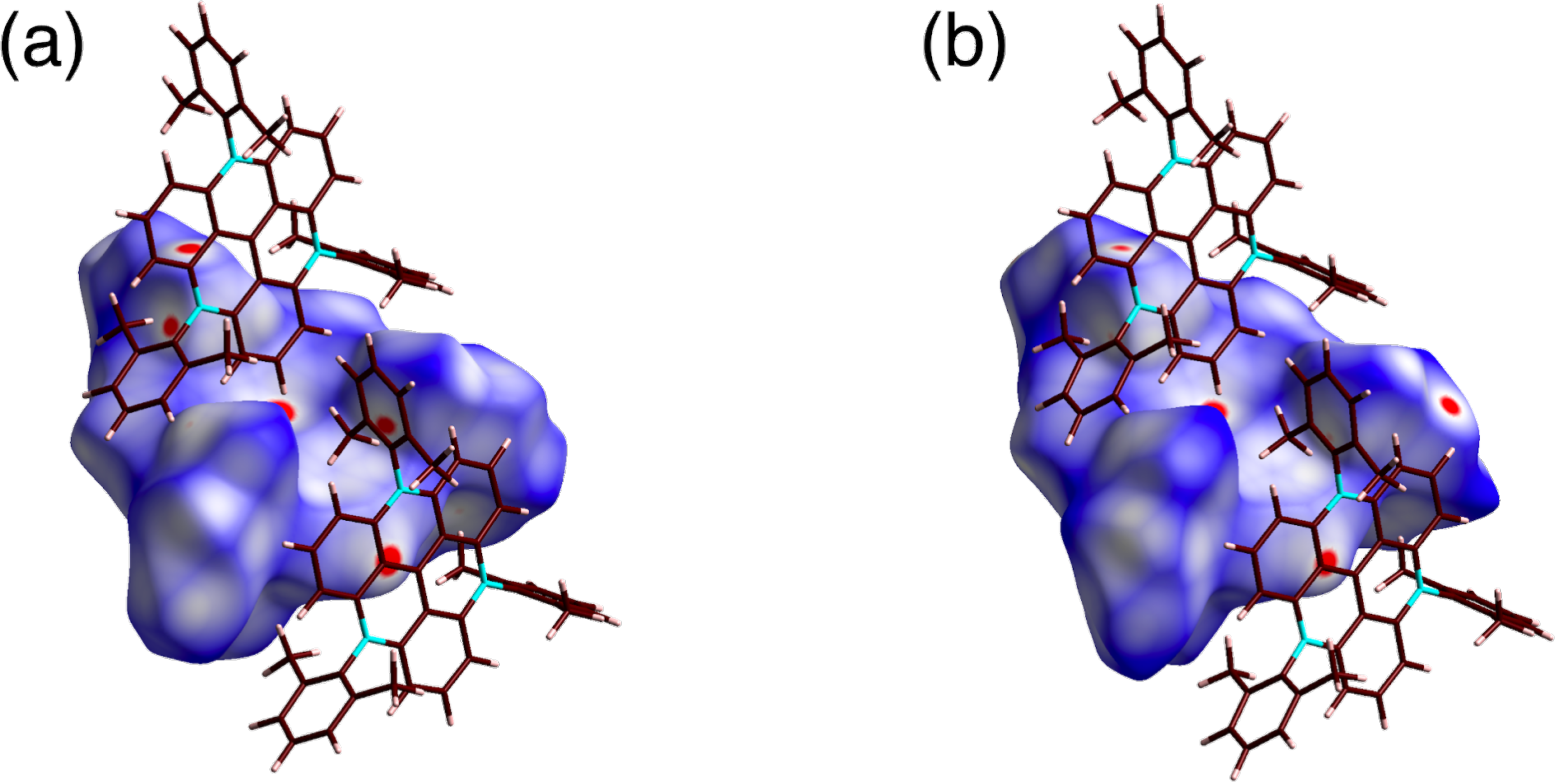
Hirshfeld surface analysis mapped with *d*_norm_ of closely contacted two **2**^+^ in (a) **2**^+^-BF_4_^−^ and (b) **2**^+^-PF_6_^−^. Atom color code: brown, pink, and blue refer to carbon, hydrogen, and nitrogen, respectively.

Solid-state ion-pairing assemblies of the ion pairs **2**^+^-X^−^ (X^−^ = Cl^−^, BF_4_^−^, PF_6_^−^, B(C_6_F_5_)_4_^−^, and PCCp^−^) were investigated by X-ray analysis of the orange rod-shaped single crystals, which were obtained by vapour diffusion using CHCl_3_/*n*-heptane for **2**^+^-Cl^−^, **2**^+^-BF_4_^−^, and **2**^+^-PCCp^−^, CH_2_Cl_2_/*n*-heptane with small amounts of MeOH and toluene for **2**^+^-PF_6_^−^, and toluene/*n*-hexane for **2**^+^-B(C_6_F_5_)_4_^−^ (Figure 3 and Figures S11–15 in Supporting Information File 1) [32]. In the single crystals, **2**^+^ showed highly planar core structures with mean-plane deviations of 0.014–0.071 Å for the 22 core atoms. 2,6-Dimethylphenyl units were orthogonally arranged with dihedral angles of 81.0°–89.9°, which are similar to those of *N*-phenyl-substituted **1b**^+^ [26]. In the packing structure, the **2**^+^-Cl^−^ units were aligned in a columnar structure with an interplane distance of 9.26 Å for the mean planes of the 22 core atoms. Such a long interplane distance is mainly derived from the bulky 2,6-dimethylphenyl units that hinder the close stacking of the TATA^+^ core units, as observed in *N*-alkyl-substituted **1a**^+^ [11–12,15–16,22] and *N*-phenyl-substituted **1b**^+^ [26]. Columnar structures with alternately arranged **2**^+^ and counteranions were also formed for **2**^+^-B(C_6_F_5_)_4_^−^ and **2**^+^-PCCp^−^ with interplane distances of 15.7 and 8.46 Å, respectively. On the other hand, in the packing structures of ion pairs **2**^+^-BF_4_^–^ and **2**^+^-PF_6_^–^, cation **2**^+^ formed herringbone structures, which were not observed in the other TATA^+^ cations that form π–π stacking structures [11–12,15–16,22]. The angles between the two staggered TATA^+^ planes were 53.7° and 51.4° for **2**^+^-BF_4_^−^ and **2**^+^-PF_6_^−^, respectively. The orthogonal arrangement of TATA^+^ and the *o*-CH_3_-substituted aryl units was suitable for forming intermolecular C–H···π interactions between the proximally located CH_3_ and TATA^+^ units, as obtained by the Hirshfeld surface analysis (Figure 4) [33–36].

**Figure 5:**
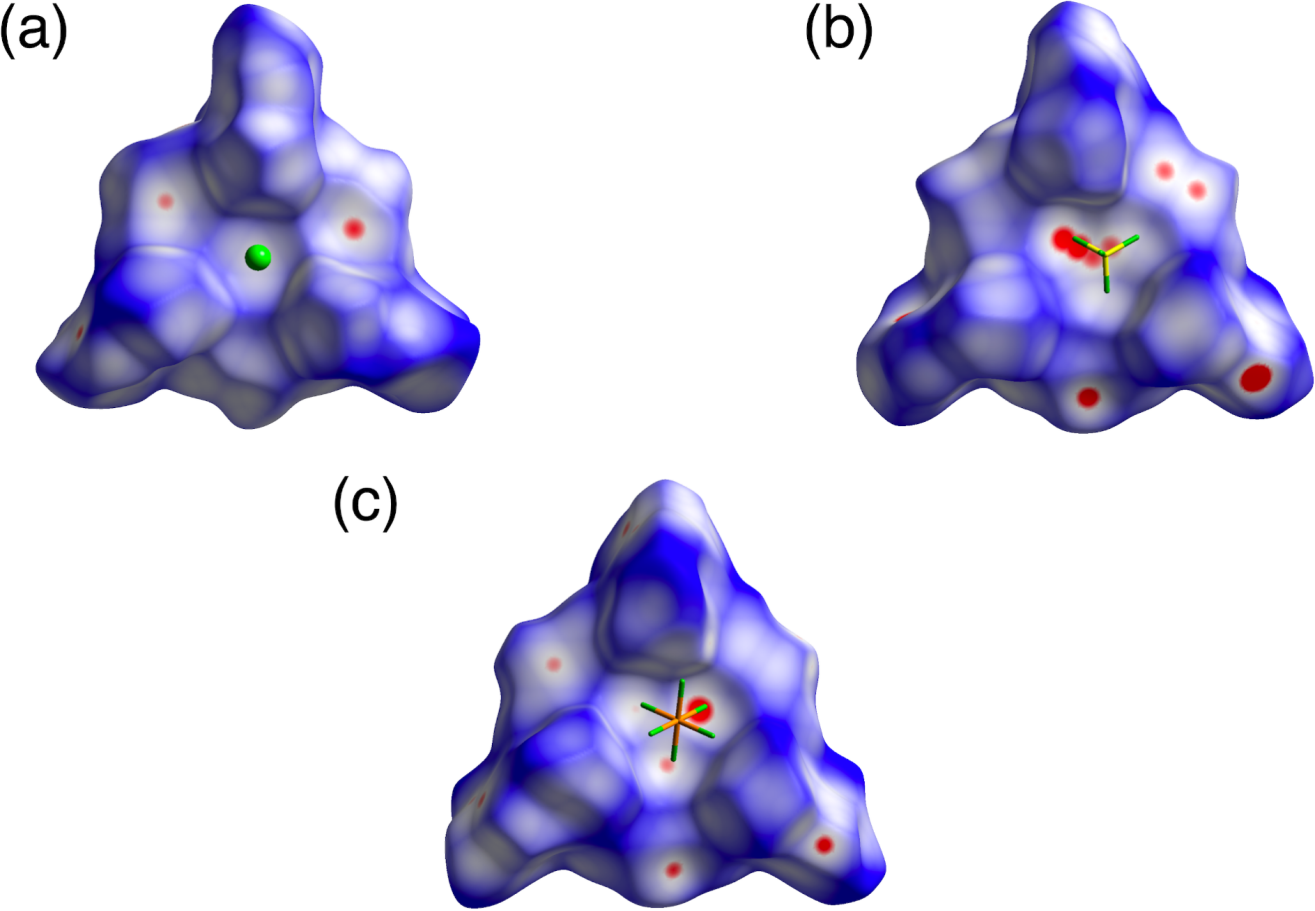
Hirshfeld surface analysis mapped with *d*_norm_ of closely contacted ion pairs: (a) **2**^+^-Cl^−^, (b) **2**^+^-BF_4_^−^, and (c) **2**^+^-PF_6_^−^. Atom color code: yellow, green, orange, and green (spherical) refer to boron, fluorine, phosphorus, and chlorine, respectively.

The arrangement of the ion pairs depended on the counteranions, which formed different interactions. Smaller counteranions were located above the electron-deficient TATA^+^ core through the interactions of the methyl units. In fact, in **2**^+^-Cl^−^, Cl^−^ was located 3.43 Å above the TATA^+^ core and was interacted with the surrounding CH_3_ units, with C(–H)···Cl^−^ distances of 3.74, 3.85, and 3.96 Å (Figure 3a, Figure 5a and Figure S17 in Supporting Information File 1). As a crystallization solvent, three CHCl_3_ molecules were located between the 2,6-dimethylphenyl units by forming hydrogen bonding with Cl^−^ with C(–H)···Cl^−^ distances of 3.36, 3.36, and 3.37 Å. A similar arrangement of the counteranions was observed for **2**^+^-BF_4_^−^ and **2**^+^-PF_6_^−^, with the BF_4_^−^ and PF_6_^−^ anions located above the TATA^+^ plane (Figure 3b,c and Figure 5b,c). Three of the fluorine atoms of BF_4_^−^ and PF_6_^−^ were pointed toward the TATA^+^ plane. The offset angles for Cl^−^, BF_4_^−^, and PF_6_^−^ to the TATA^+^ central carbon were 87.7°, 78.1°, and 84.3°, respectively. The distances between fluorine and the TATA^+^ mean plane in **2**^+^-BF_4_^−^ were 2.94, 3.09, and 3.14 Å, while those in **2**^+^-PF_6_^−^ were 2.90, 3.17, and 3.20 Å. Hirshfeld surface analysis of the proximally located ion pairs mapped with *d*_norm_ clearly indicated close contacts between the anions and the TATA^+^ plane (Figure 5b,c and Figures S18 and S19 in Supporting Information File 1).

**Figure 6:**
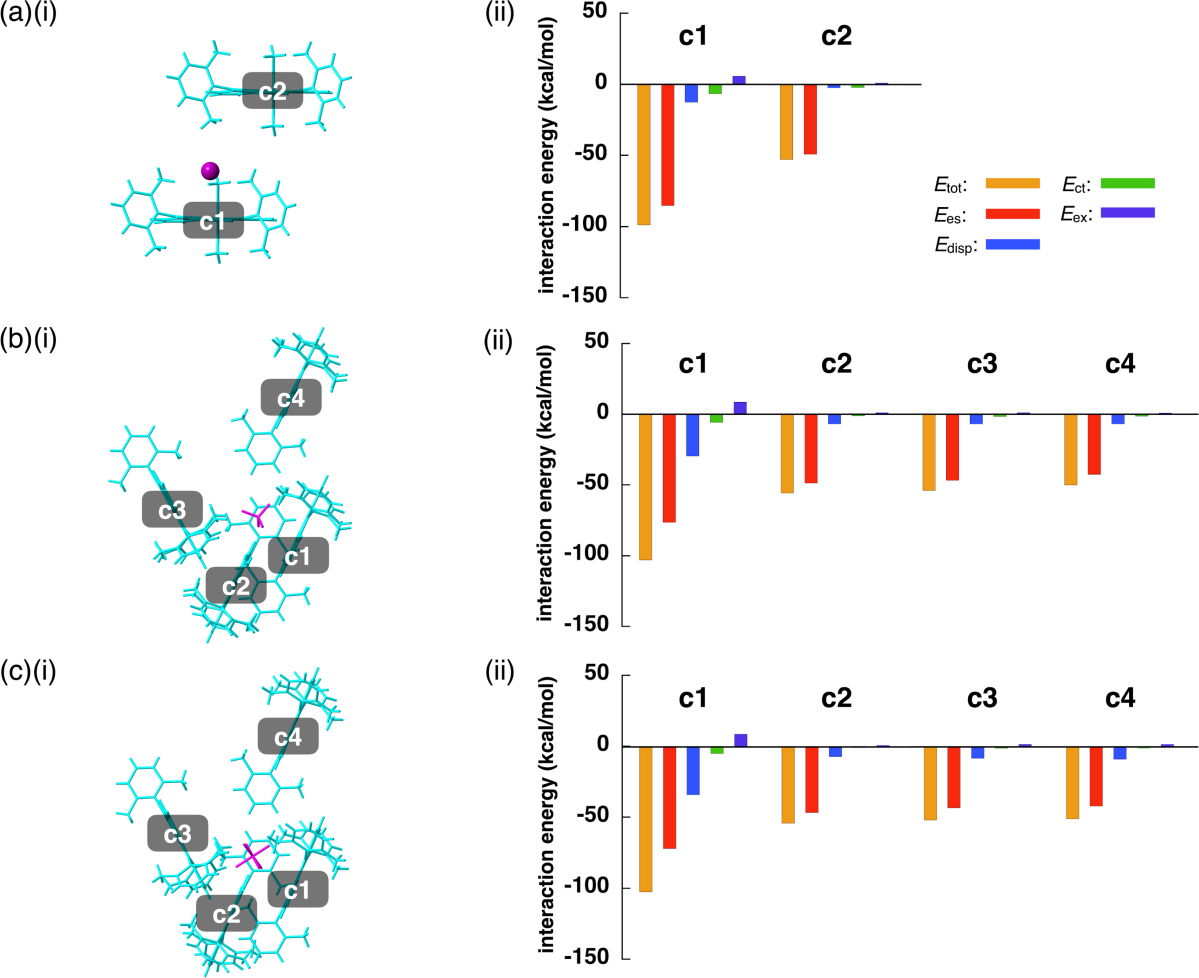
(i) Single-crystal X-ray structures and (ii) interaction energies for the pairs (a) **2**^+^-Cl^−^, (b) **2**^+^-BF_4_^−^, and (c) **2**^+^-PF_6_^−^. In (i), the cation and the anion are represented in cyan and magenta colors, respectively.

Theoretically performed energy decomposition analysis (EDA) based on FMO2-MP2 using the basis set of NOSeC-V-DZP with MCP [37–40] for the anion and closely located **2**^+^, as suggested by Hirshfeld surface analysis, revealed that short counteranion–TATA^+^ plane distances resulted mainly from electrostatic interaction. Closely contacted ion pairs for **2**^+^-Cl^−^, **2**^+^-BF_4_^−^, and **2**^+^-PF_6_^−^ showed total energies (*E*_tot_) of −98.1, −102.2, and −101.9 kcal/mol, respectively, with the main contribution from electrostatic forces (*E*_es_) of −84.6, −75.6, and −71.2 kcal/mol, and dispersion forces (*E*_disp_) of −12.5, −29.5, and −34.2 kcal/mol, respectively (Figure 6a–c and Figures S26–28 in Supporting Information File 1). Other sets of the ion pairs in the crystal structures showed smaller interaction energies. For example, the pair of **2**^+^ and BF_4_^−^ located at the side of TATA^+^ showed the *E*_tot_, *E*_es_, and *E*_disp_ values of −55.7, −48.6, and −6.9 kcal/mol, respectively, which are smaller than those of the ion pair contacting the TATA^+^ plane.

**Figure 7:**
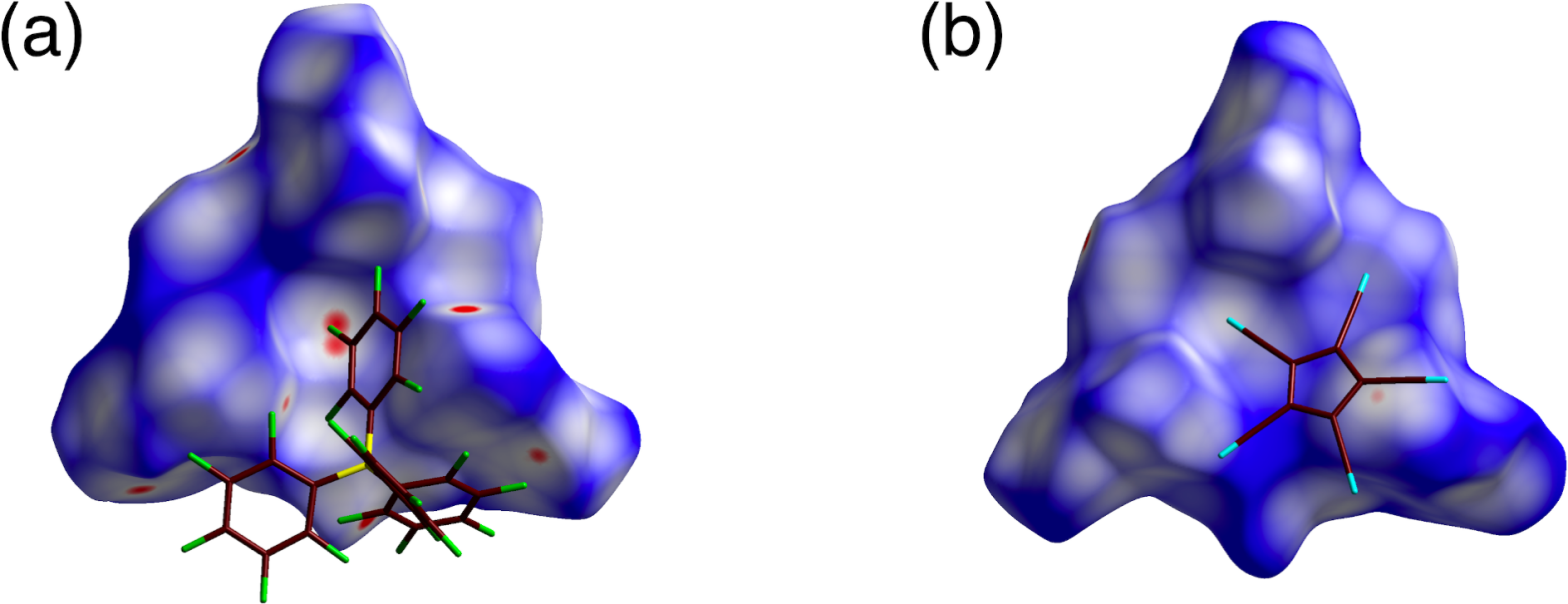
Hirshfeld surface analysis mapped with *d*_norm_ of closely contacted ion pairs: (a) **2**^+^-B(C_6_F_5_)_4_^−^ and (b) **2**^+^-PCCp^−^. Atom color code: brown, yellow, blue, and green refer to carbon, boron, nitrogen, and fluorine, respectively.

**Figure 8:**
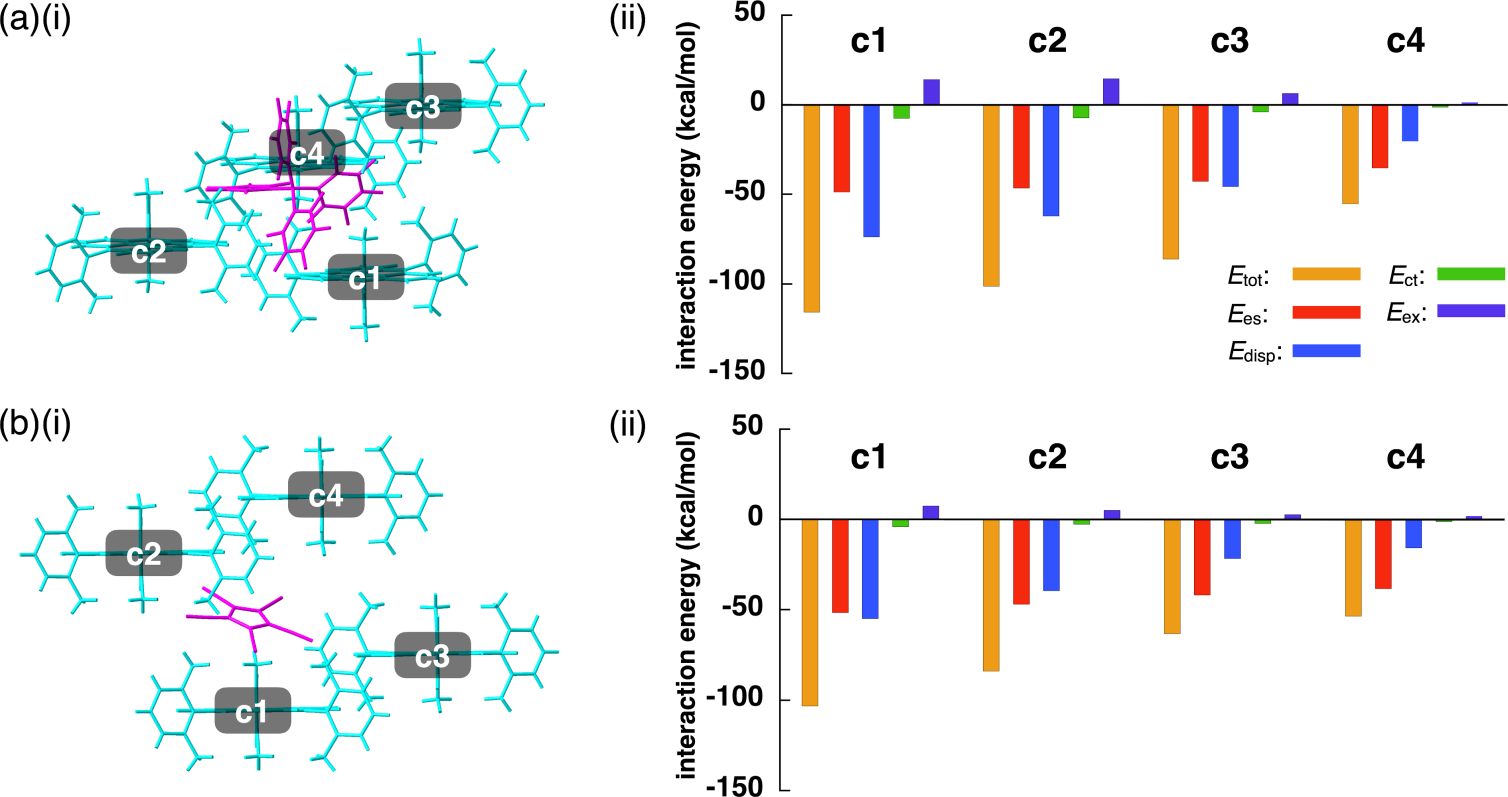
(i) Single-crystal X-ray structures and (ii) interaction energies for the pairs for (a) **2**^+^-B(C_6_F_5_)_4_^−^ and (b) **2**^+^-PCCp^−^. In (i), the cation and the anion are represented in cyan and magenta colors, respectively.

Ion pairs of larger counteranions formed ion-pairing assemblies in which the counteranions were displaced from the top of the TATA^+^ planes. In **2**^+^-B(C_6_F_5_)_4_^−^, the distance between the boron of B(C_6_F_5_)_4_^−^ and the central carbon of TATA^+^ was 7.32 Å, with an offset angle of 47.1° (Figure 3d, Figure 7a, and Figures S14 and S20 in Supporting Information File 1). On the other hand, in **2**^+^-PCCp^−^, the PCCp^−^ unit (centroid of the five-membered ring) was located 5.55 Å from the central carbon of TATA^+^ with an offset angle of 69.4° (Figure 3e, Figure 7b, and Figures S15 and S21 in Supporting Information File 1). The PCCp^−^ unit was inclined at 29.9° to the TATA^+^ plane, with the closest distance of 3.31 Å between the cyano-N and TATA^+^ mean plane. Two proximally located PCCp^−^ units formed a partially stacked dimer with a stacking distance of 3.43 Å. The B(C_6_F_5_)_4_^−^ and PCCp^−^ ion pairs with the nearest set of ion pairs showed the *E*_tot_ values of −115.1 and −100.7 kcal/mol, respectively, with *E*_es_/*E*_disp_ values of −48.6/−73.7 and −51.5/−54.9 kcal/mol, respectively (Figure 8a,b and Figures S29 and S30 in Supporting Information File 1). The energy values for **2**^+^-PCCp^−^ are larger than other sets of ion pairs located at the side of TATA^+^ showing *E*_tot_ of −63.1 kcal/mol. On the other hand, another proximally located ion pair in **2**^+^-B(C_6_F_5_)_4_^−^ showed similar *E*_tot_, *E*_es_, and *E*_disp_ values of −101.2, −46.4, and −62.2 kcal/mol, respectively, indicating that the bulkier counteranion interacted with several surrounding cations. Smaller *E*_es_ values for **2**^+^-B(C_6_F_5_)_4_^−^ and **2**^+^-PCCp^−^ compared to those for the ion pairs comprising smaller counteranions can be ascribed to the larger distances between **2**^+^ and the counteranions owing to steric hindrance.

## Conclusion

In this study, *N*-(2,6-dimethylphenyl)-substituted TATA^+^ was used as a bulky cation to combine with anions of different sizes for forming ion pairs. In the solid state, the bulky TATA^+^ cation formed ion-pairing assemblies without stacking of the TATA^+^ planes owing to the steric hindrance of the bulky *N*-substituents, indicating that the arrangement of the TATA^+^ cation was largely dependent on the coexisting counteranions. In particular, the TATA^+^ cation in the BF_4_^−^ and PF_6_^−^ ion pairs formed herringbone structures by the aid of intermolecular C–H···π interactions. Such characteristic anion-driven assemblies of TATA^+^ are not known, and further studies on ion-pairing assemblies of modified TATA^+^ can lead to the development of functional materials, comprising multiple components associated via *^i^*π–*^i^*π interactions that are the interactions between charged (ionic) π-systems, with interesting electronic properties like electric conductivities.

## Experimental

### General procedures

Starting materials were purchased from FUJIFILM Wako Pure Chemical Corp., Nacalai Tesque Inc., and Sigma-Aldrich Co. and were used without further purification unless otherwise stated. According to the previous procedure [30], 4,8,12-tris(2,6-dimethylphenyl)-4,8,12-triazatriangulenium as a Cl^−^ ion pair, **2**^+^-Cl^−^, was prepared. NMR spectra used in the characterization of products were recorded on a JEOL ECA-600 600 MHz spectrometer. All NMR spectra were referenced to residual solvent. UV–visible absorption spectra were recorded on a Hitachi U-4500 spectrometer. Fluorescence spectra were recorded on a Hitachi F-4500 fluorescence spectrometer. MALDI–TOF MS was recorded on a Shimadzu Axima-CFRplus. TLC analyses were carried out on aluminum sheets coated with silica gel 60 (Merck 5554). Column chromatography was performed on Wakogel C-300.

### Preparation of ion pairs

**4,8,12-Tris(2,6-dimethylphenyl)-4,8,12-triazatriangulenium as a BF_4_**^−^** ion pair, 2^+^-BF_4_**^−^**.** According to the literature procedure [26], to a Schlenk tube were added 2,6-dimethylaniline (7.0 mL, 59 mmol) and tris(2,6-dimethoxyphenyl)methylium as a Cl^−^ ion pair [22] (504 mg, 1.09 mmol). The Schlenk tube was placed in an oil bath and was heated from rt to reflux over a period of 30 min. The reaction mixture was allowed to reach rt, and then the aniline was removed by short silica gel column chromatography (Wakogel C-300, eluent: MeOH/CH_2_Cl_2_ with an increment of MeOH). The residue was chromatographed over ion-exchanged resin (Amberlite IRA402BL, eluent: MeOH/CH_2_Cl_2_) and solvents were removed by evaporation. Then, to a MeOH solution (150 mL) of the solid was added AgBF_4_ (269 mg, 1.38 mmol), and the reaction mixture was stirred at rt for 10 min, followed by filtration, and evaporation to dryness. The residue was purified by silica gel column chromatography (Wakogel C-300; eluent: MeOH/AcOEt/CH_2_Cl_2_ 1:2:8) and was recrystallized from CH_2_Cl_2_/*n*-hexane to afford **2**^+^-BF_4_^−^ (143 mg, 0.209 mmol, 19%) as a red solid. *R*_f_ 0.33 (MeOH/EtOAc/CH_2_Cl_2_ 1:2:8); ^1^H NMR (600 MHz, CDCl_3_, 20 °C) δ (ppm) 7.68 (t, *J* = 8.4 Hz, 3H, TATA-H), 7.51 (t, *J* = 7.8 Hz, 3H, Ar-H), 7.44 (d, *J* = 7.8 Hz, 6H, Ar-H), 6.37 (d, *J* = 8.4 Hz, 6H, TATA-H), 2.10 (s, 18H, CH_3_); ^13^C NMR (151 MHz, CDCl_3_, 20 °C) δ (ppm) 142.15, 140.66, 138.90, 136.12, 134.80, 130.88, 130.80, 110.29, 106.31, 17.63; ^19^F NMR (564 MHz, CDCl_3_, 20 °C) δ (ppm) −157.91 (s, ^10^BF_4_^−^), −157.96 (s, ^11^BF_4_^−^); UV–vis (CH_3_CN) λ_max_, nm (ε, 10^5^ M^−1^ cm^−1^): 272 (1.28), 337 (0.06), 349 (0.09), 524 (0.21); MALDI–TOF MS (*m/z*) (% intensity): (positive) 594.3 (100), 595.3 (40), 596.3 (10), [M − BF_4_]^+^ calcd for C_43_H_36_N_3_, 594.29; (negative) 86.0 (20), 87.0 (100); [M – C_43_H_36_N_3_]^−^ calcd for BF_4_, 87.00. This compound was further characterized by single-crystal X-ray diffraction analysis.

**4,8,12-Tris(2,6-dimethylphenyl)-4,8,12-triazatriangulenium as a PF_6_^− ^ion pair, 2^+^-PF_6_^−^.** To a MeOH solution (3.0 mL) of **2**^+^-BF_4_^−^ (4.98 mg, 7.3 μmol) was added NaPF_6_ (3.68 mg, 22 μmol), and the reaction mixture was stirred at rt for 15 min, followed by filtration and evaporation to dryness. The residue was purified by silica gel column chromatography (Wakogel C-300; eluent: 5% MeOH/CH_2_Cl_2_) and was recrystallized from CH_2_Cl_2_/*n*-hexane to afford **2**^+^-PF_6_^−^ (3.68 mg, 5.0 μmol, 68%) as a red solid. *R*_f_ 0.40 (5% MeOH/CH_2_Cl_2_); ^1^H NMR (600 MHz, CDCl_3_, 20 °C) δ (ppm) 7.63 (t, *J* = 8.4 Hz, 3H, TATA-H), 7.50 (t, *J* = 7.8 Hz, 3H, Ar-H), 7.43 (d, *J* = 7.8 Hz, 6H, Ar-H), 6.35 (d, *J* = 8.4 Hz, 6H, TATA-H), 2.11 (s, 18H, CH_3_); ^13^C NMR (151 MHz, CDCl_3_, 20 °C) δ (ppm) 142.40, 140.77, 138.59, 136.46, 135.03, 130.73, 130.69, 110.66, 106.15, 17.61; ^19^F NMR (564 MHz, CDCl_3_, 20 °C) δ (ppm) −77.26 (d, *J* = 712 Hz, 6F); UV–vis (CH_3_CN), λ_max_, nm (ε, 10^5^ M^−1^ cm^−1^): 272 (1.30), 337 (0.07), 349 (0.09), 523 (0.21); MALDI–TOF MS (*m/z*) (% intensity): (positive) 594.3 (100), 595.3 (40), 596.3 (10), [M − F_6_P]^+^ calcd for C_43_H_36_N_3_, 594.29; (negative) 145.1 (100), [M − C_49_H_30_N_3_]^−^ calcd for F_6_P, 144.96. This compound was further characterized by single-crystal X-ray diffraction analysis.

**4,8,12-Tris(2,6-dimethylphenyl)-4,8,12-triazatriangulenium as a B(C_6_F_5_)_4_^− ^ion pair, 2^+^-B(C_6_F_5_)_4_^−^.** To a CH_2_Cl_2_ solution (3.0 mL) of **2**^+^-BF_4_^−^ (4.82 mg, 7.1 μmol) was added the Li^+^ salt of tetrakis(pentafluorophenyl)borate (LiB(C_6_F_5_)_4_, 12.6 mg, 18 μmol), and the reaction mixture was stirred at rt for 30 min, followed by filtration and evaporation to dryness. The residue was purified by silica gel column chromatography (Wakogel C-300; eluent: 5% MeOH/CH_2_Cl_2_) and was recrystallized from CH_2_Cl_2_/*n*-hexane to afford **2**^+^-B(C_6_F_5_)_4_^−^ (3.89 mg, 3.1 μmol, 44%) as a red solid. *R*_f_ 0.74 (5% MeOH/CH_2_Cl_2_); ^1^H NMR (600 MHz, CDCl_3_, 20 °C) δ (ppm) 7.68 (t, *J* = 8.4 Hz, 3H, TATA-H), 7.51 (t, *J* = 7.8 Hz, 3H, Ar-H), 7.44 (d, *J* = 7.2 Hz, 6H, Ar-H), 6.38 (d, *J* = 8.4 Hz, 6H, TATA-H), 2.11 (s, 18H, CH_3_); ^13^C NMR (151 MHz, CDCl_3_, 20 °C) δ (ppm) 148.27 (d, *J*_13C–19F_ = 242 Hz), 142.18, 140.70, 138.92, 138.22 (d, *J*_13C–19F_ = 241 Hz), 135.98, 136.31 (d, *J*_13C–19F_ = 246 Hz), 134.76, 131.00, 130.84, 124.30, 110.24, 106.33, 17.37; ^19^F NMR (564 MHz, CDCl_3_, 20 °C) δ (ppm) −135.76 (s, 8F, Ar-F), −166.67 (t, *J* = 21.4 Hz, 4F, Ar-F), −170.25 (t, *J* = 18.0 Hz, 8F, Ar-F); UV–vis (CH_3_CN, λ_max_, nm (ε, 10^5^ M^−1^ cm^−1^): 272 (1.26), 337 (0.07), 349 (0.10), 524 (0.20); MALDI–TOF MS (*m/z)* (% intensity): (positive) 594.3 (100), 595.3 (40), 596.3 (10), [M − C_24_BF_20_]^+^ calcd for C_43_H_36_N_3,_ 594.29; (negative) 678.0 (20), 679.0 (100), 680.0 (20), [M – C_43_H_36_N_3_]^–^ calcd for C_24_BF_20_, 678.98. This compound was further characterized by single-crystal X-ray diffraction analysis.

**4,8,12-Tris(2,6-dimethylphenyl)-4,8,12-triazatriangulenium as a PCCp^− ^ion pair, 2^+^-PCCp^−^.** To a MeOH solution (3.0 mL) of **2**^+^-BF_4_^−^ (4.88 mg, 7.2 μmol) was added sodium pentacyanocyclopentadienide [18–19] (NaPCCp, 4.74 mg, 22 μmol), and the reaction mixture was stirred at rt for 10 min, followed by filtration and evaporation to dryness. The residue was purified by silica gel column chromatography (Wakogel C-300; eluent: 5% MeOH/CH_2_Cl_2_) and was recrystallized from CH_2_Cl_2_/*n*-hexane to afford **2**^+^-PCCp^−^ (3.21 mg, 4.1 μmol, 57%) as a red solid. *R*_f_ 0.56 (5% MeOH/CH_2_Cl_2_); ^1^H NMR (600 MHz, CDCl_3_, 20 °C) δ (ppm) 7.68 (t, *J* = 8.4 Hz, 3H, TATA-H), 7.53 (t, *J* = 7.8 Hz, 3H, Ar-H), 7.45 (d, *J* = 7.8 Hz, 6H, Ar-H), 6.39 (d, *J* = 8.4 Hz, 6H, TATA-H), 2.09 (s, 18H, CH_3_); ^13^C NMR (151 MHz, CDCl_3_, 20 °C) δ (ppm) 142.24, 140.72, 138.96, 136.04, 134.80, 131.00, 130.87, 113.57, 110.34, 106.37, 102.64, 17.65; UV–vis (CH_3_CN, λ_max_, nm (ε, 10^5^ M^−1^ cm^−1^): 272 (1.33), 337 (0.07), 349 (0.10), 524 (0.20); MALDI–TOF MS (*m/z*) (% intensity): (positive) 594.3 (100), 595.3 (40), 596.3 (10), [M − C_10_N_5_]^+^ calcd for C_43_H_36_N_3_, 594.29; (negative) 190.1 (100), 191.1 (10), [M − C_43_H_36_N_3_]^−^ calcd for C_10_N_5_, 190.02. This compound was further characterized by single-crystal X-ray diffraction analysis.

### Method for single-crystal X-ray analysis

Crystallographic data are summarized in Table 1. A single crystal of **2**^+^-Cl^−^ was obtained by vapor diffusion of *n*-heptane into a CHCl_3_ solution. The data crystal was a red prism of approximate dimensions 0.20 mm × 0.02 mm × 0.01 mm. A single crystal of **2**^+^-BF_4_^−^ was obtained by vapor diffusion of *n*-heptane into a CHCl_3_ solution. The data crystal was a red prism of approximate dimensions 0.30 mm × 0.10 mm × 0.05 mm. A single crystal of **2**^+^-PF_6_^−^ was obtained by vapor diffusion of *n*-hexane into a CH_2_Cl_2_ with small amounts of MeOH and toluene. The data crystal was a red prism of approximate dimensions 0.20 mm × 0.02 mm × 0.01 mm. A single crystal of **2**^+^-PCCp^−^ was obtained by vapor diffusion of *n*-heptane into a CHCl_3_ solution. The data crystal was a red prism of approximate dimensions 0.050 mm × 0.030 mm × 0.010 mm. A single crystal of **2**^+^-B(C_6_F_5_)_4_^−^ was obtained by vapor diffusion of *n*-hexane into a toluene solution. The data crystal was a red prism of approximate dimensions 0.05 mm × 0.01 mm × 0.01 mm. The data of **2**^+^-Cl^−^ and **2**^+^-BF_4_^−^ were collected at 90 K on a Bruker D8 Venture diffractometer with Mo Kα radiation (λ = 0.71073 Å) focused by a multilayer confocal mirror, whereas the data of **2**^+^-PF_6_^−^ and **2**^+^-B(C_6_F_5_)_4_^−^ were collected at 100 K on a DECTRIS EIGER X 1M diffractometer with Si(111) monochromated synchrotron radiation (λ = 0.8125 and 0.8120 Å, respectively) at BL40XU (SPring-8) [41–42]. The data of **2**^+^-PCCp^−^ was collected at 90 K on a Rigaku Pliatus 3 CdTe 1M with Si(311) monochromated synchrotron radiation (λ = 0.4144 Å) at BL02B1 (SPring-8) [43]. All the structures were solved by dual-space method. The structures were refined by a full-matrix least-squares method by using a SHELXL 2014 [44] (Yadokari-XG) [45–46]. In each structure, the non-hydrogen atoms were refined anisotropically. CIF files (CCDC-2364292–2364296) can be obtained free of charge from the Cambridge Crystallographic Data Centre via https://www.ccdc.cam.ac.uk/data_request/cif.

**Table 1:** Crystallographic details.

	**2**^+^-Cl^−^	**2**^+^-BF_4_^−^	**2**^+^-PF_6_^−^	**2**^+^-B(C_6_F_5_)_4_^−^	**2**^+^-PCCp^−^

formula	C_43_H_36_N_3_Cl· 3CHCl_3_	C_43_H_36_N_3_BF_4_· 2CHCl_3_	C_43_H_36_N_3_·F_6_P	C_43_H_36_N_3_·C_24_BF_20_· C_7_H_8_·0.5C_6_H_14_	C_43_H_36_N_3_·C_10_N_5_· CHCl_3_
fw	988.30	920.29	739.72	1409.02	904.26
crystal size, mm	0.20 × 0.02 × 0.01	0.30 × 0.10 × 0.05	0.20 × 0.02 × 0.01	0.05 × 0.01 × 0.01	0.050 × 0.030 × 0.010
crystal system	triclinic	monoclinic	monoclinic	triclinic	triclinic
space group	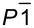 (no. 2)	*P*2_1_/*n* (no. 14)	*P*2_1_/*n* (no. 14)	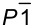 (no. 2)	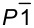 (no. 2)
*a*, Å	10.3193(6)	19.3043(12)	15.1182(2)	14.6519(5)	11.079(10)
*b*, Å	12.8955(8)	11.7445(6)	11.95190(10)	15.4923(6)	14.418(12)
*c*, Å	18.2429(12)	19.3182(12)	19.4163(2)	15.9933(5)	16.284(15)
α, °	78.520(2)	90	90	99.408(3)	90.562(7)
β, °	78.621(2)	103.358(2)	91.4290(10)	99.229(3)	105.230(8)
γ, °	76.892(2)	90	90	108.879(3)	102.795(7)
*V*, Å^3^	2287.4(2)	4261.3(4)	3507.26(7)	3299.2(2)	2441(4)
*ρ*_calcd_, g cm^–3^	1.435	1.434	1.401	1.418	1.230
*Z*	2	4	4	2	2
*T*, K	90(2)	90(2)	100(2)	100(2)	90(2)
μ, mm^–1^	0.646^a^	0.458^a^	0.209^b^	0.167^b^	0.065^b^
no. of reflns	39674	81192	41635	35844	20901
no. of unique reflns	7912	8668	8216	14804	9959
variables	559	566	485	908	592
λ, Å	0.71073^a^	0.71073^a^	0.8125^b^	0.8120^b^	0.4144^b^
*R*_1_ (*I* > 2*σ*(*I*))	0.0708	0.0470	0.0395	0.0737	0.0577
*wR*_2_ (*I* > 2*σ*(*I*))	0.1633	0.1218	0.1142	0.2198	0.1738
*GOF*	1.061	1.016	1.050	1.058	0.963

^a^Mo Kα. ^b^Synchrotron radiation.

**DFT calculations.** DFT calculations for the geometrical optimizations were carried out by using the *Gaussian 16* program [31].

## Supporting Information

File 1 ^1^H, ^13^C, and ^19^F NMR spectra of new ion pairs, details for crystal structures, and theoretical calculations.

## Data Availability

All data that supports the findings of this study is available in the published article and/or the supporting information to this article.
