# Supplementary material for: Anion-dependent ion-pairing assemblies of triazatriangulenium cation that interferes with stacking structures
Source: Beilstein J Org Chem. 2024 Oct 10;20:2567–76. doi: 10.3762/bjoc.20.215 (PMC11472656; doi:10.3762/bjoc.20.215)
Supplement: File 1 — 1H, 13C, and 19F NMR spectra of new ion pairs, details for crystal structures, and theoretical calculations. [file Beilstein_J_Org_Chem-20-2567-s001.pdf]

BEILSTEIN JOURNAL OF ORGANIC CHEMISTRY

Supporting Information

for

Anion-dependent ion-pairing assemblies of triazatriangulenium cation that interferes with stacking structures

Yohei Haketa, Takuma Matsuda and Hiromitsu Maeda

Beilstein J. Org. Chem. **2024,** *20,* 2567–2576. doi:10.3762/bjoc.20.215

^{1}H, ^{13}C, and ^{19}F NMR spectra of new ion pairs, details for crystal structures, and theoretical calculations

License and Terms: This is a supporting information file under the terms of the Creative Commons Attribution License (https://creativecommons.org/licenses/by/4.0). Please note that the reuse, redistribution and reproduction in particular requires that the author(s) and source are credited and that individual graphics may be subject to special legal provisions.
The license is subject to the *Beilstein Journal of Organic Chemistry* terms and conditions: (https://www.beilstein-journals.org/bjoc/terms)

Table of contents

1. Spectroscopic data S2

Figure S1–4 ^{1}H and ^{13}C NMR spectra S2
Figure S5 UV–vis absorption spectra S9

2. X-ray crystallographic data S10

Figure S6–10 Ortep drawings S10
Figure S11–16 Packing diagrams S14
Figure S17–21 Hirshfeld analysis S17

3. Theoretical studies S19

Figure S22 Optimized structure S19
Figure S23 ESP mapping S19
Figure S24,25 Molecular orbitals and TD-DFT calculations S20
Figure S26–30 EDA analyses of single-crystal X-ray structures S22
Cartesian coordination of optimized structures S27

1. Spectroscopic data

Figure S1 (a) ^{1}H NMR, (b) ^{13}C NMR, and (c) ^{19}F NMR spectra of **2**$^{+}$-BF_4^{-} in $CDCl_3$.

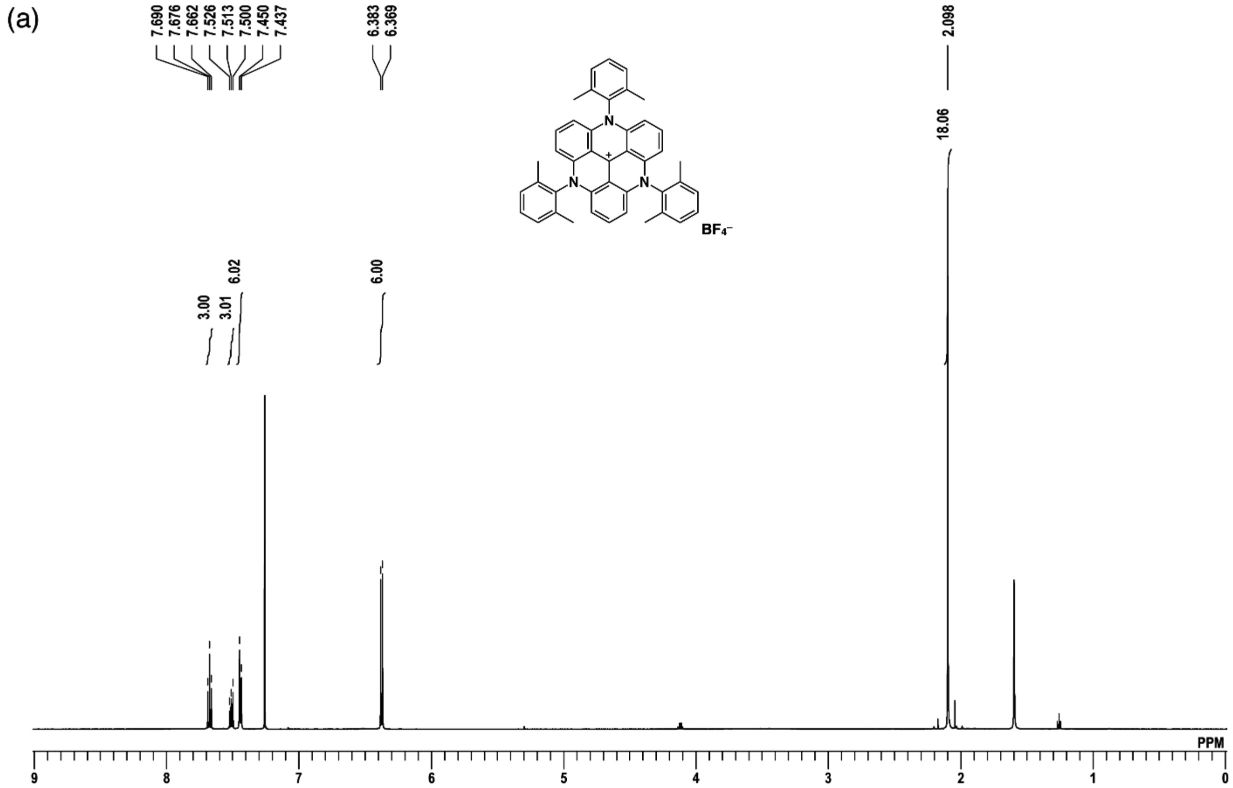

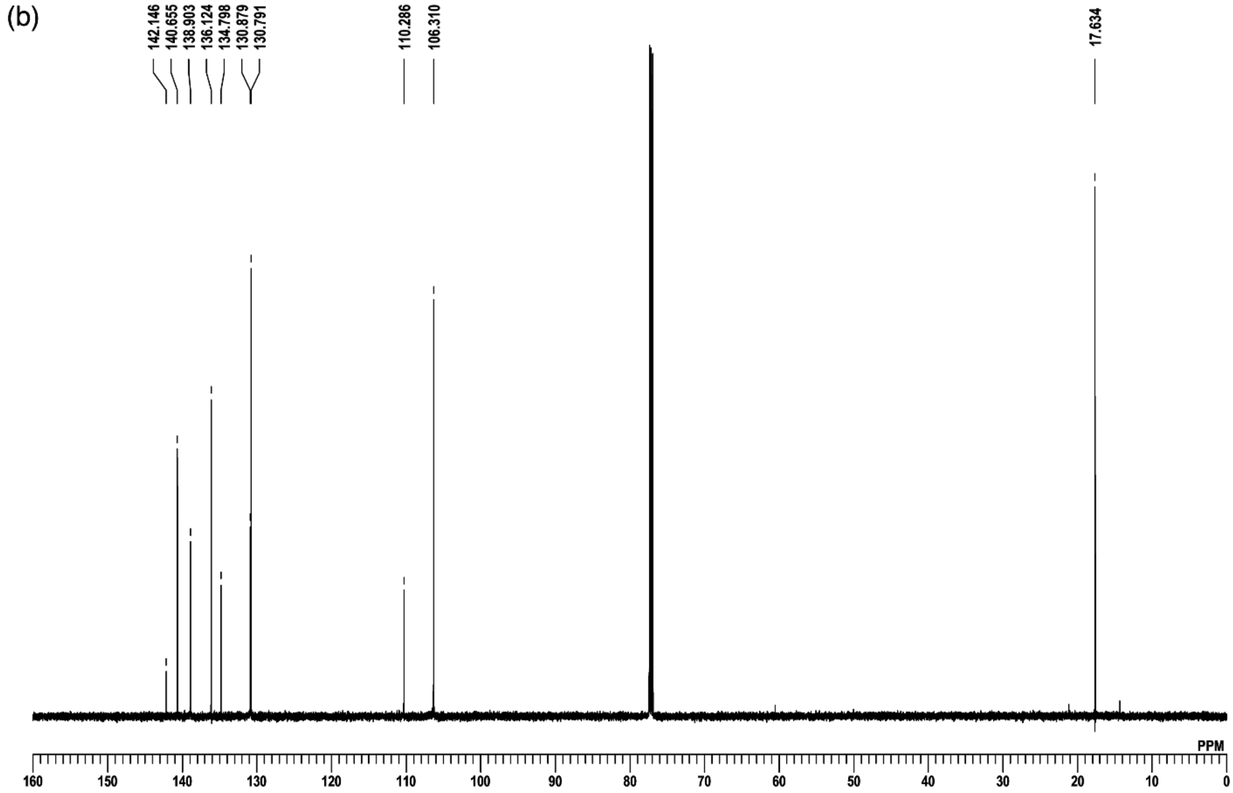

(c)

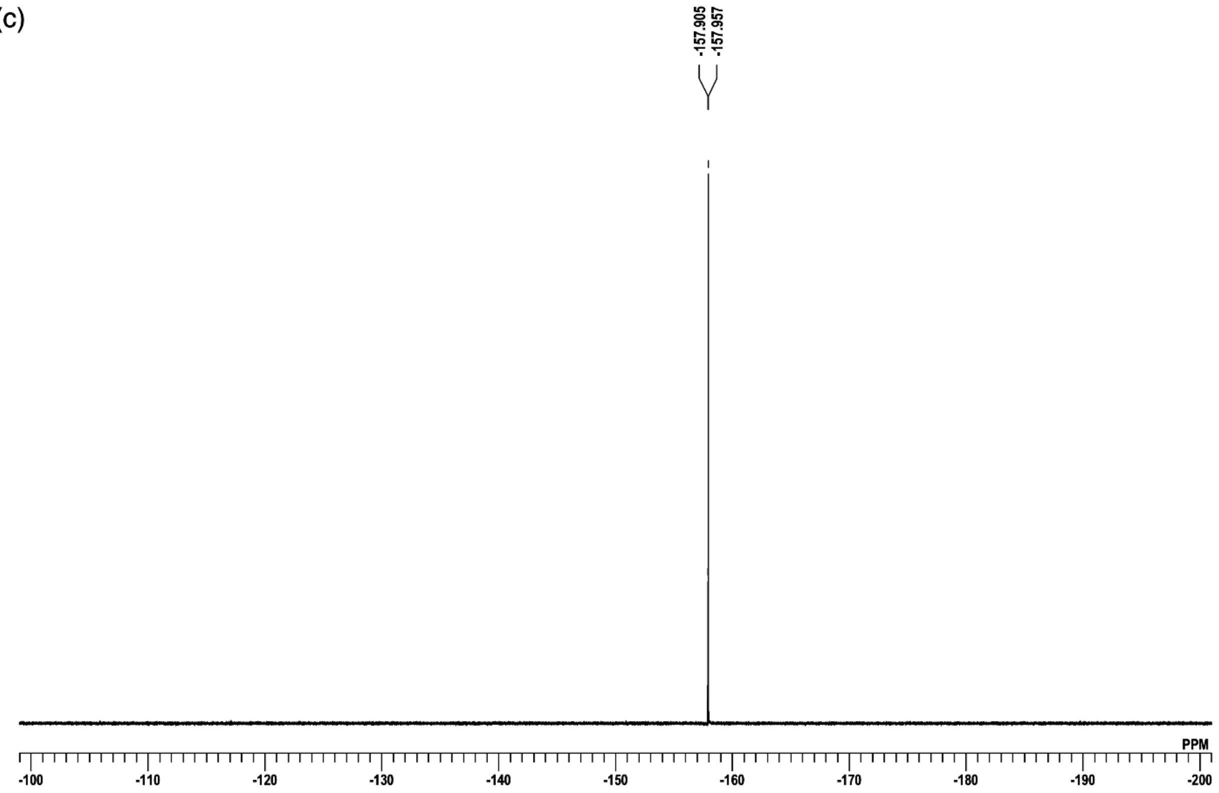

Figure S2 (a) ^{1}H NMR, (b) ^{13}C NMR, and (c) ^{19}F NMR spectra of **2**$^{+}$-PF_6^{-} in $CDCl_3$.

(a)

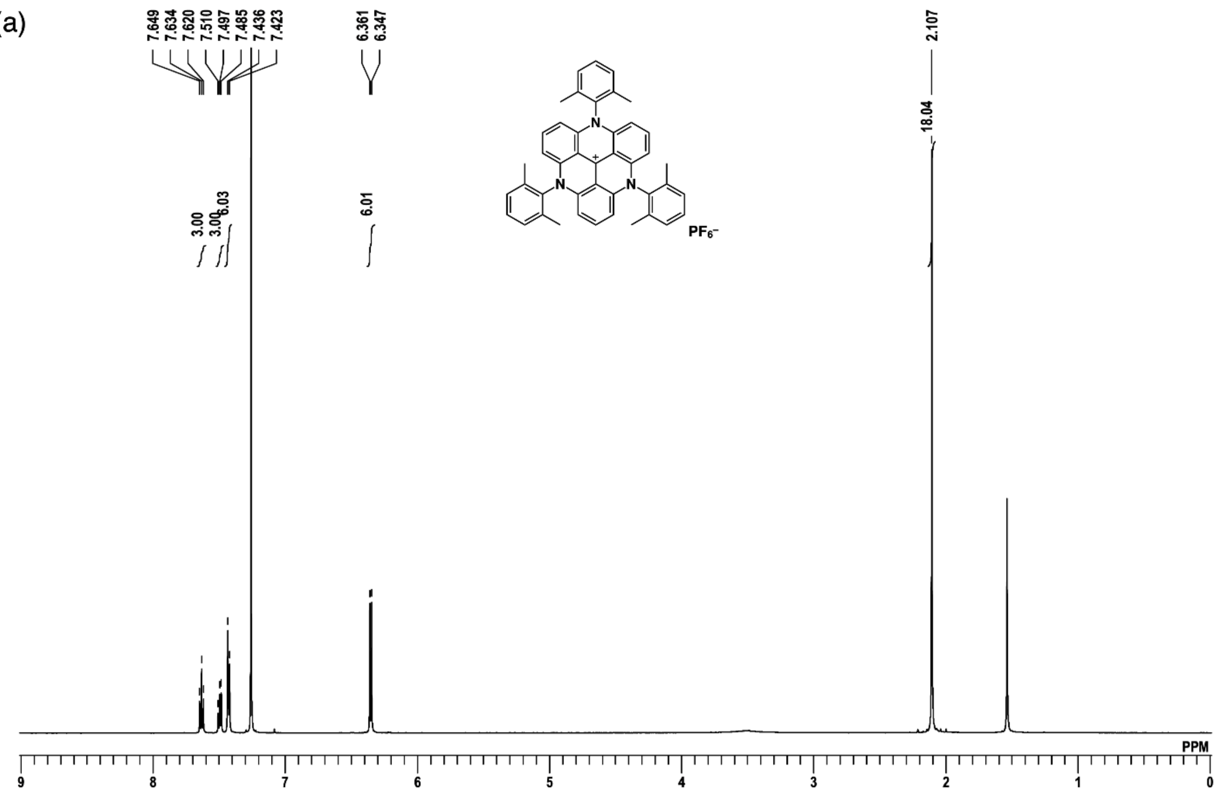

(b)

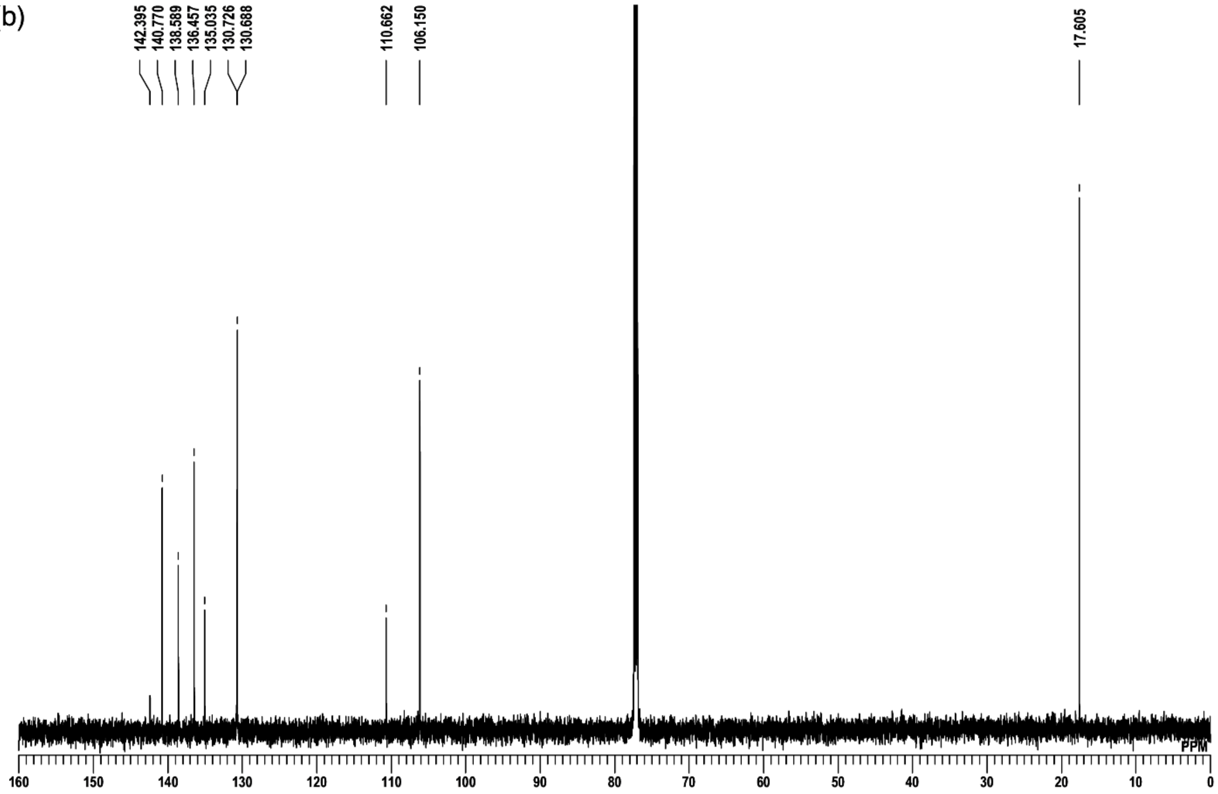

(c)

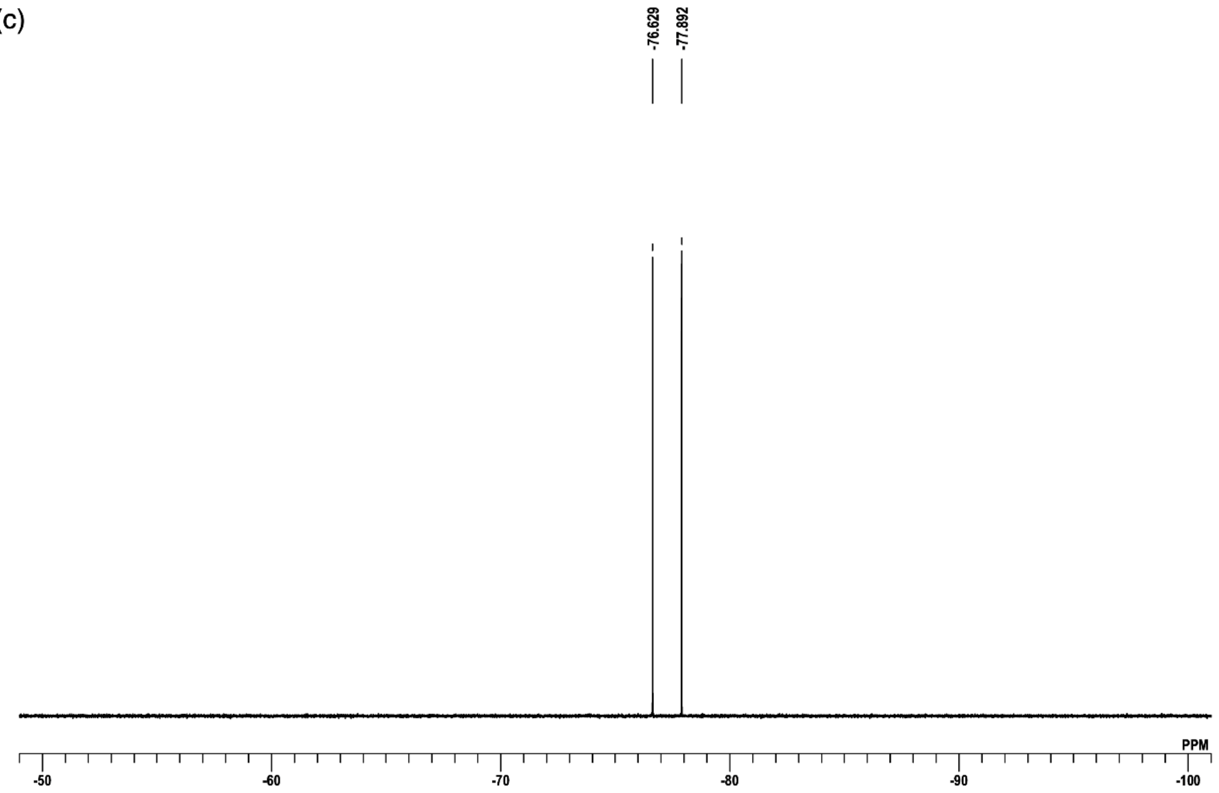

Figure S3 (a) ^{1}H NMR, (b) ^{13}C NMR, and (c) ^{19}F NMR spectra of **2**$^{+}$-$B(C_6F_5)_4^{-}$ in $CDCl_3$.

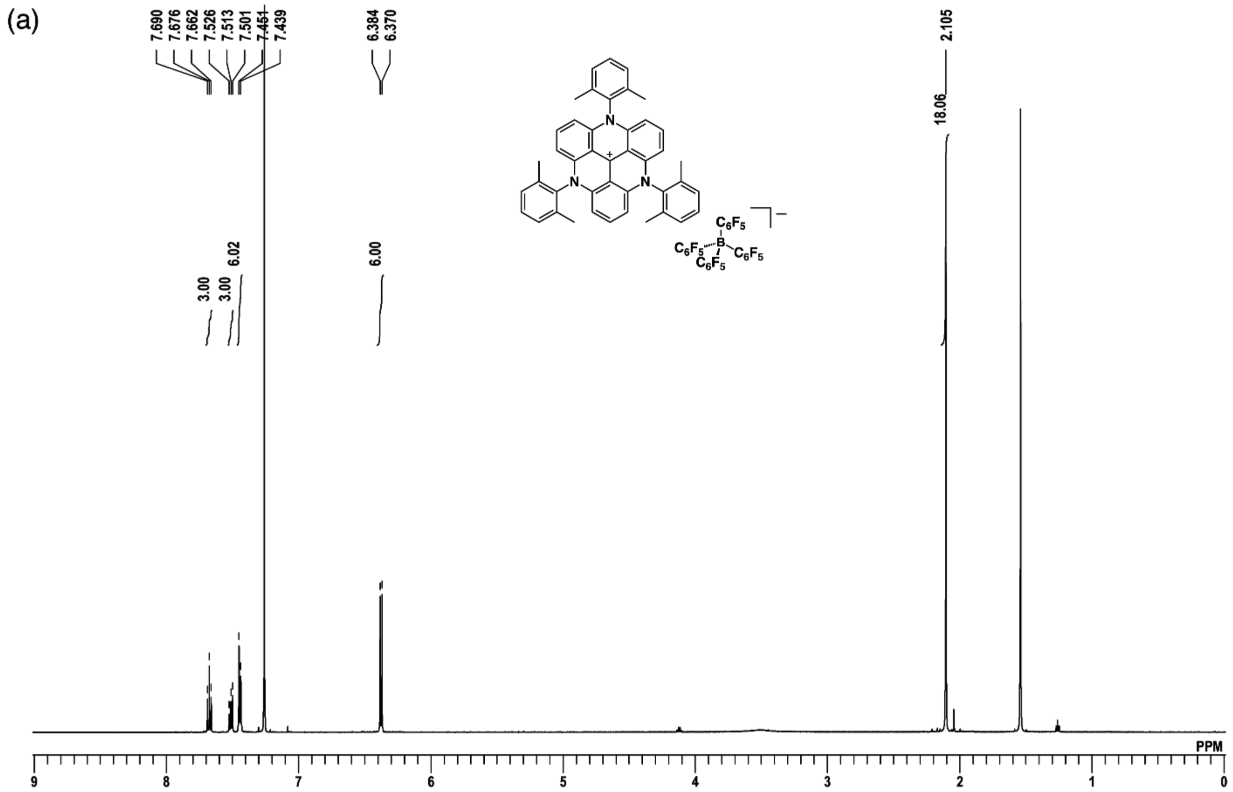

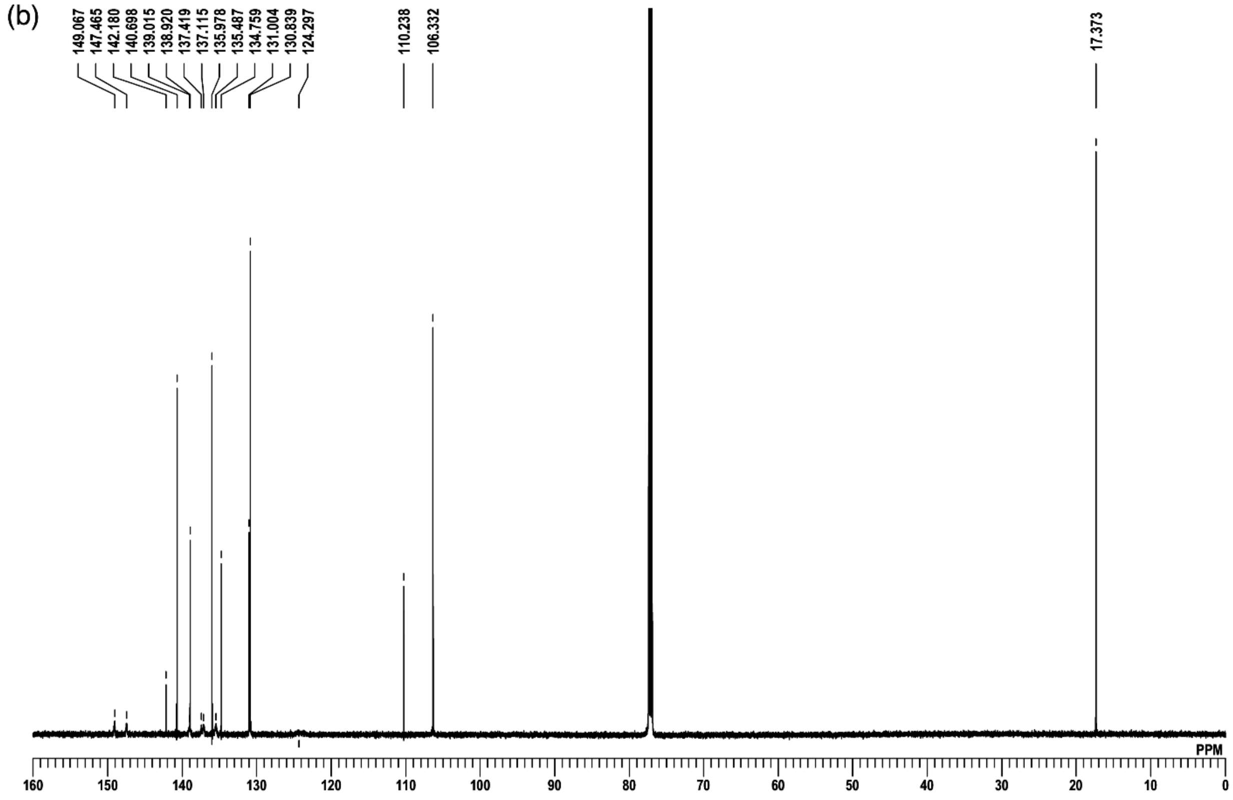

(c)

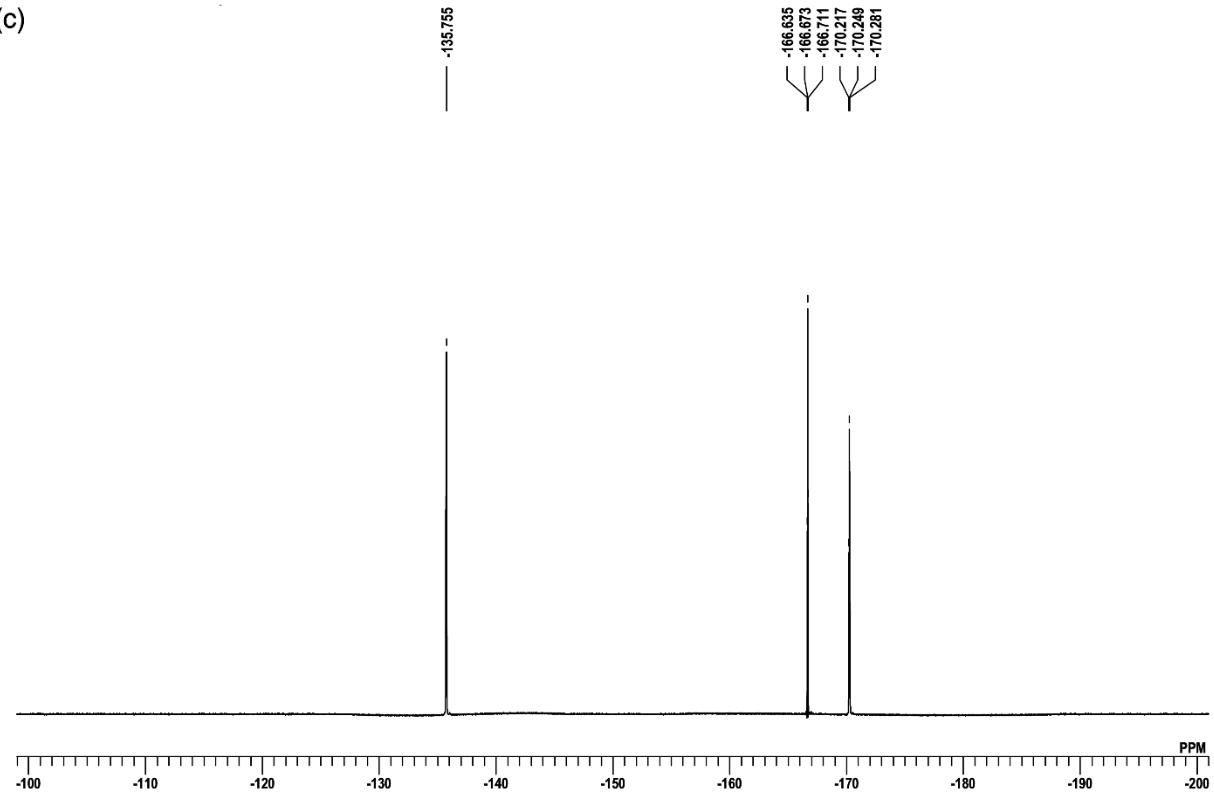
-135.755
-166.635
-166.673
-166.711
-170.217
-170.249
-170.281
PPM
-100
-110
-120
-130
-140
-150
-160
-170
-180
-190
-200

Figure S4 (a) ^{1}H NMR and (b) ^{13}C NMR spectra of **2**$^+$-PCCp$^-$ in CDCl$_3$.

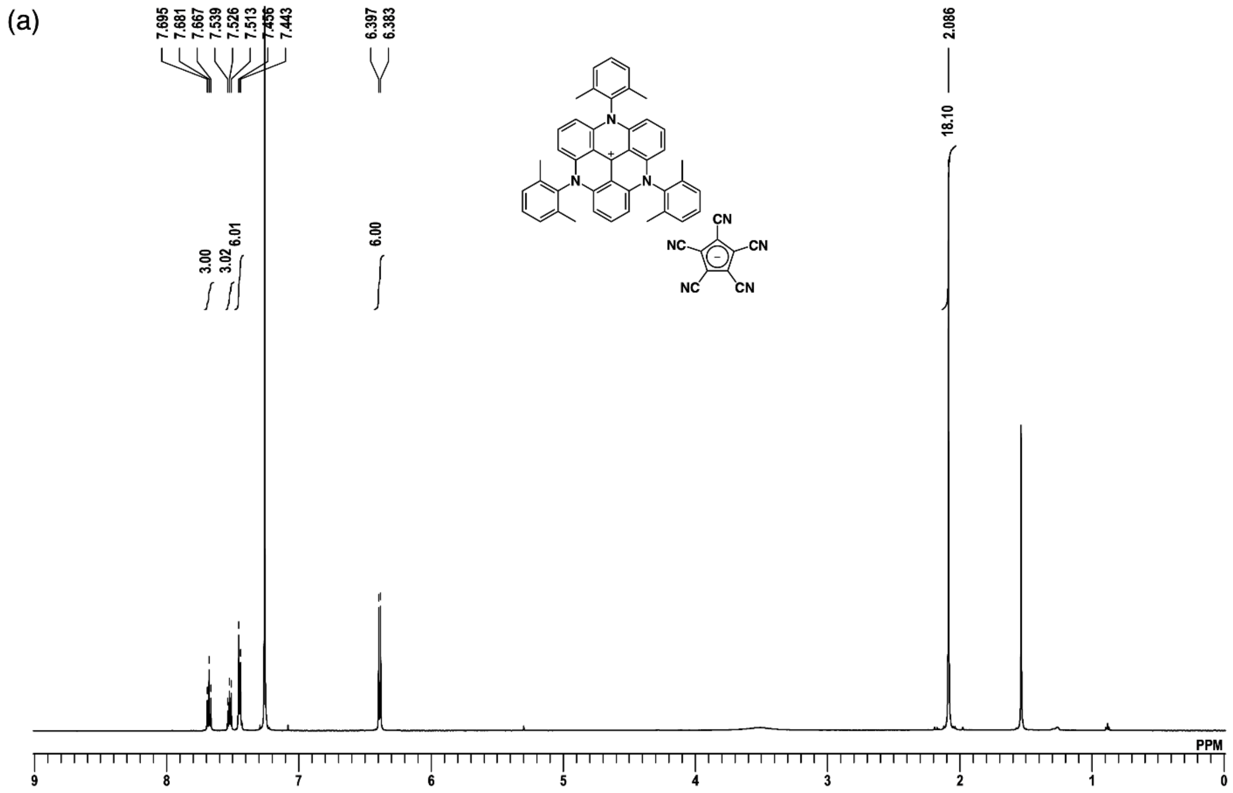

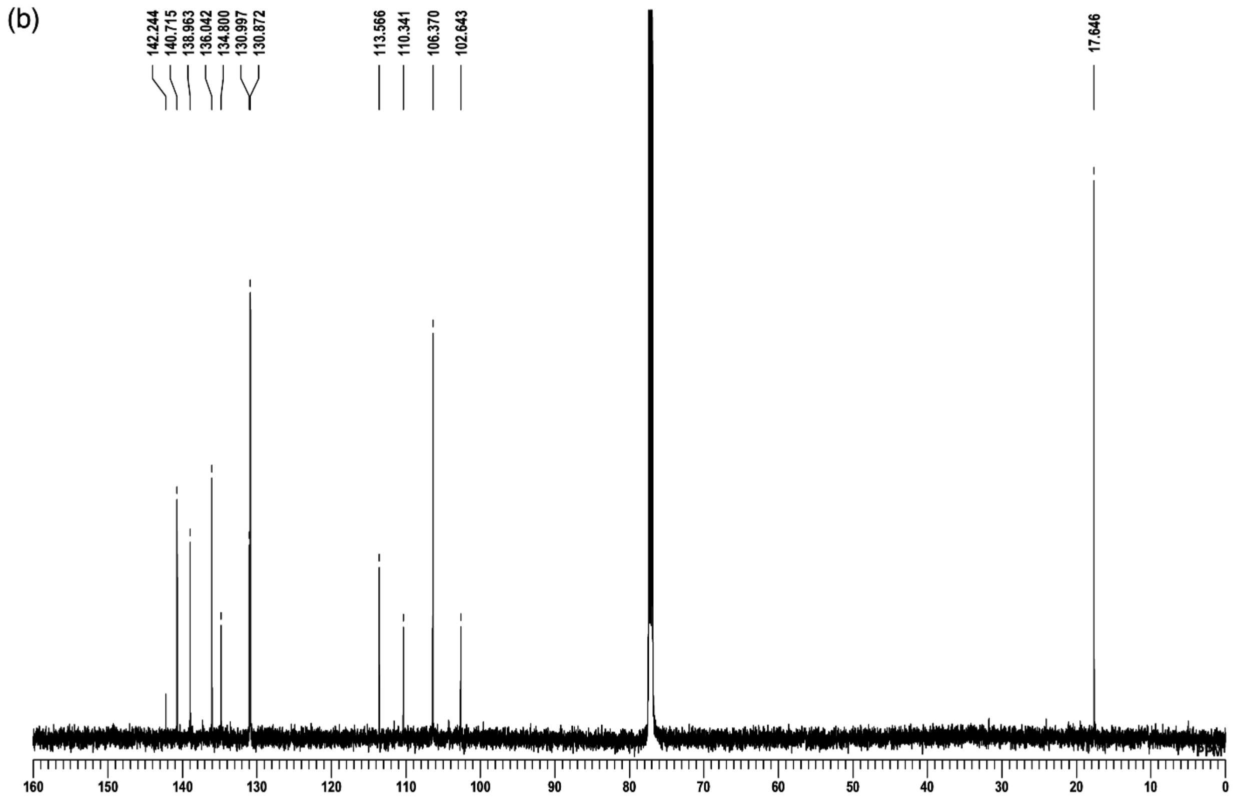

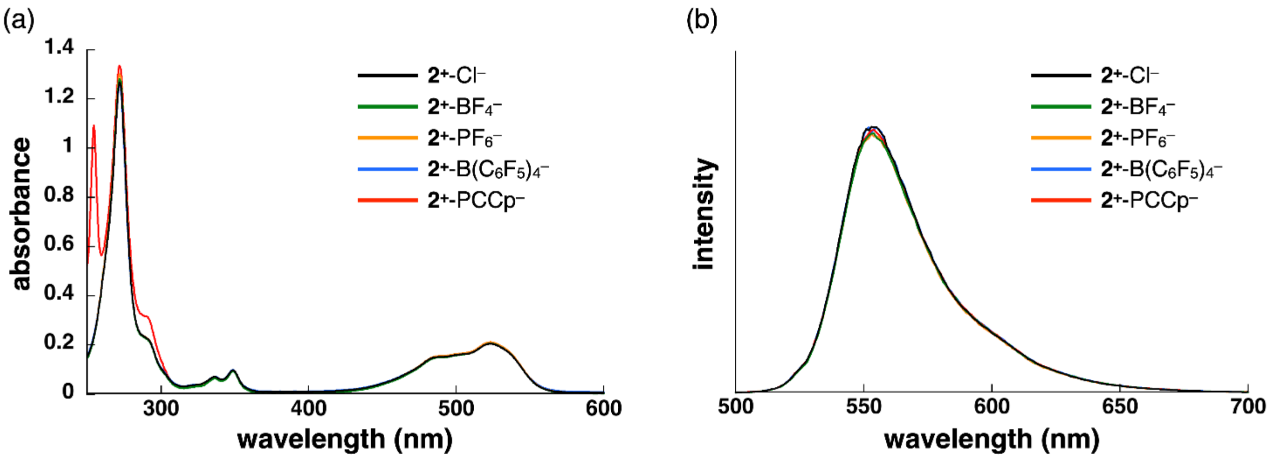

Figure S5 (a) UV–vis absorption and (b) fluorescence spectra of $\mathbf{2}^{+}$-X^{-} (X^{-} = Cl^{-},[S1] BF_4^{-}, PF_6^{-}, $B(C_6F_5)_4^{-}$, $PCCp^{-}$) in CH_3CN (1×10^{-5} M) upon excitation at 524 nm for (b).

[S1] Li, D.; Silveira, O. J.; Matsuda, T.; Hayashi, H.; Maeda, H.; Foster, A. S.; Kawai, S. *Angew. Chem. Int. Ed*. **2024**, *63*, e202411893.

2. X-ray crystallographic data

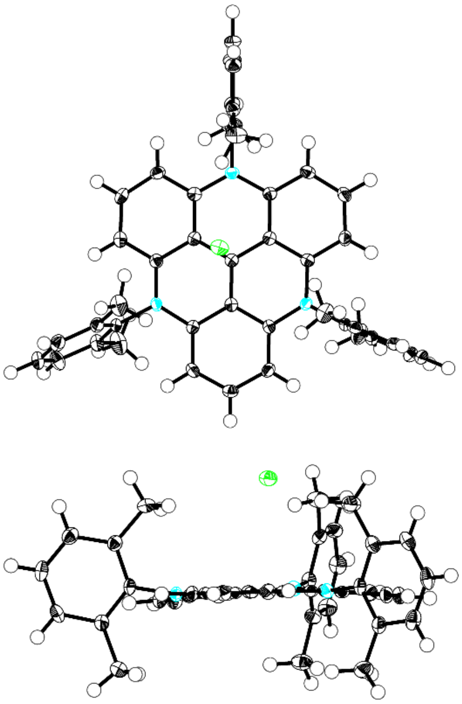

Figure S6 Ortep drawing of single-crystal X-ray structure (top and side views) of **2**$^{+}$-Cl^{-}. Thermal ellipsoids are scaled to the 50% probability level. Solvent molecules are omitted for clarity. Atom code: black, white (sphere), blue, and green refer to carbon, hydrogen, nitrogen, and chlorine, respectively.

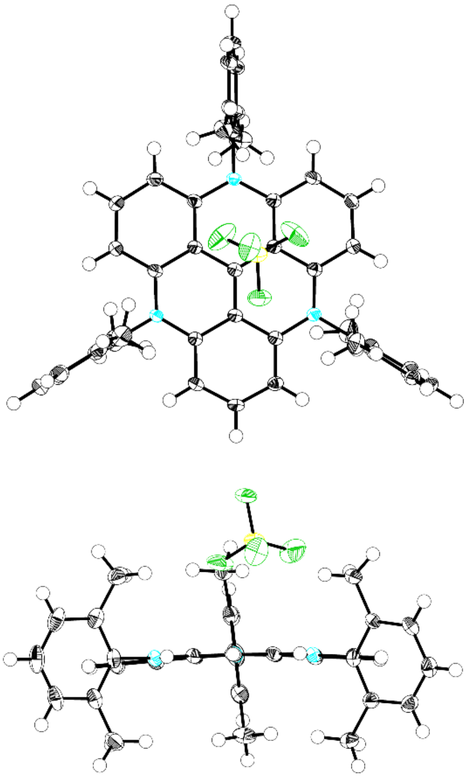

Figure S7 Ortep drawing of single-crystal X-ray structure (top and side views) of **2**$^{+}$-BF_4^{-}. Thermal ellipsoids are scaled to the 50% probability level. Solvent molecules are omitted for clarity. Atom code: black, white (sphere), yellow, blue, and green refer to carbon, hydrogen, boron, nitrogen, and fluorine, respectively.

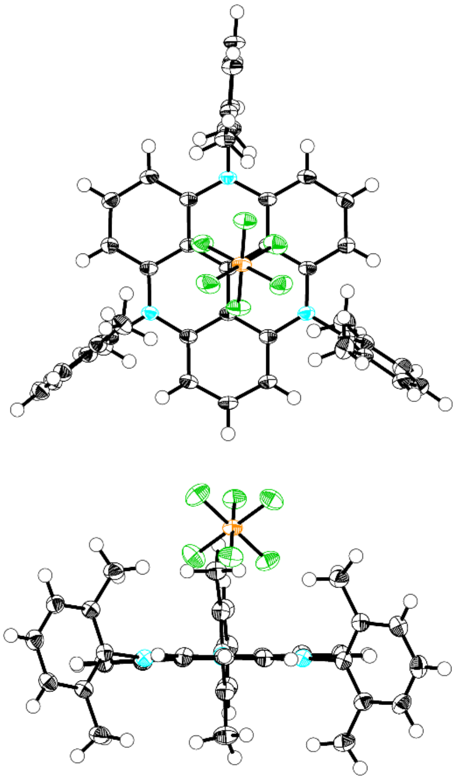

Figure S8 Ortep drawing of single-crystal X-ray structure (top and side views) of **2**$^{+}$-PF_6^{-}. Thermal ellipsoids are scaled to the 50% probability level. Atom code: black, white (sphere), blue, green, and tangerine refer to carbon, hydrogen, nitrogen, fluorine, and phosphorus, respectively.

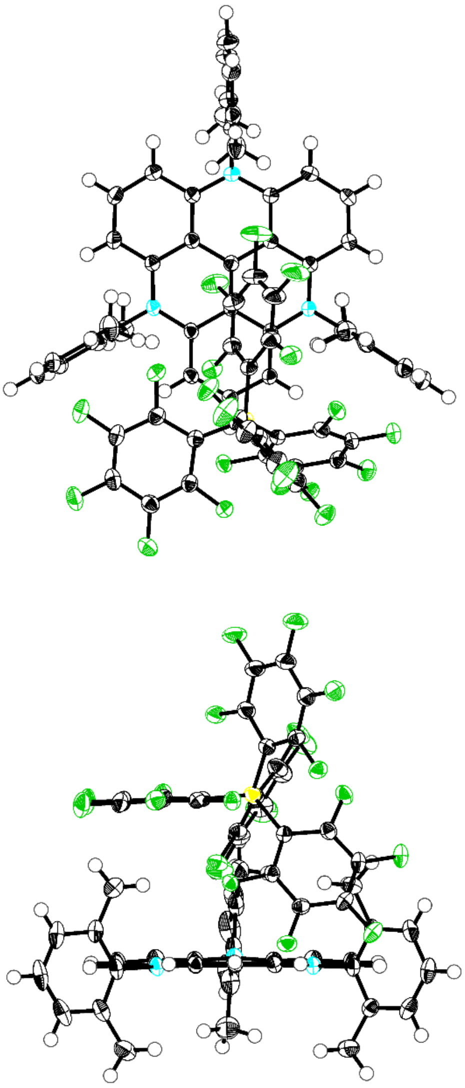

Figure S9 Ortep drawing of single-crystal X-ray structure (top and side views) of **2**$^+$-B(C_6F_5)$_4^-$. Thermal ellipsoids are scaled to the 50% probability level. Solvent molecules are omitted for clarity. Atom code: black, white (sphere), yellow, blue, and green refer to carbon, hydrogen, boron, nitrogen, and fluorine, respectively.

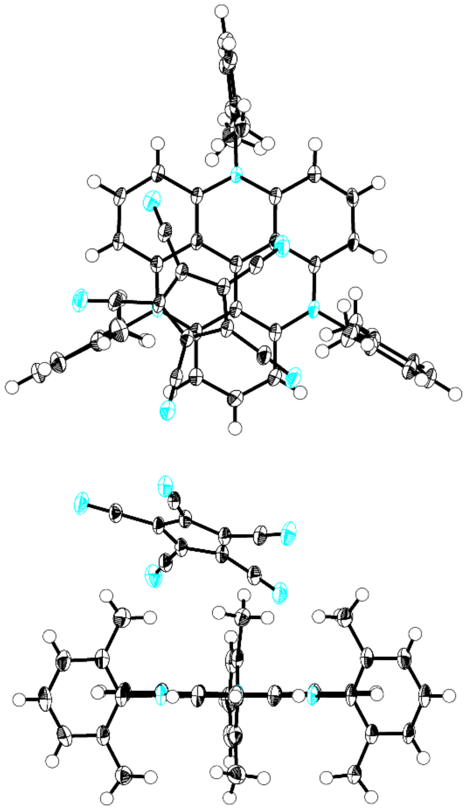

Figure S10 Ortep drawing of single-crystal X-ray structure (top and side views) of **2**$^{+}$-PCCp^{-}. Thermal ellipsoids are scaled to the 50% probability level. Solvent molecules are omitted for clarity. Atom code: black, white (sphere), and blue refer to carbon, hydrogen, and nitrogen, respectively.

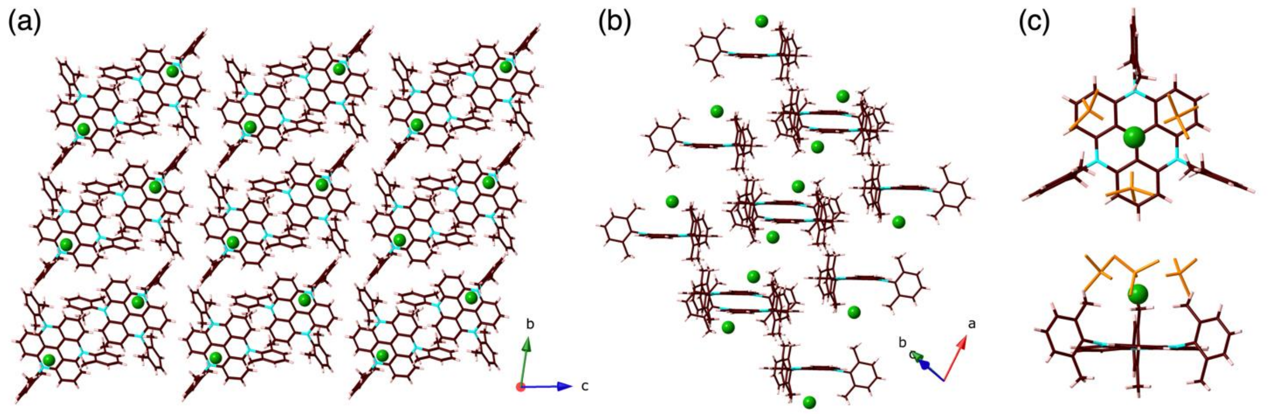

Figure S11 Single-crystal X-ray structure of **2**$^{+}$-Cl^{-}: (a) top and (b) side views of the packing structure, and (c) monomers. Solvent molecules are omitted for clarity in (a) and (b). In (c), Cl^{-} formed hydrogen bonding with surrounding three $CHCl_3$ molecules (indicated as orange color). The dihedral angles between the TATA^{+} plane and N-aryl groups were 81.0°, 87.3°, and 89.9°. Atom color code: brown, pink, blue, and green refer to carbon, hydrogen, nitrogen, and chlorine, respectively.

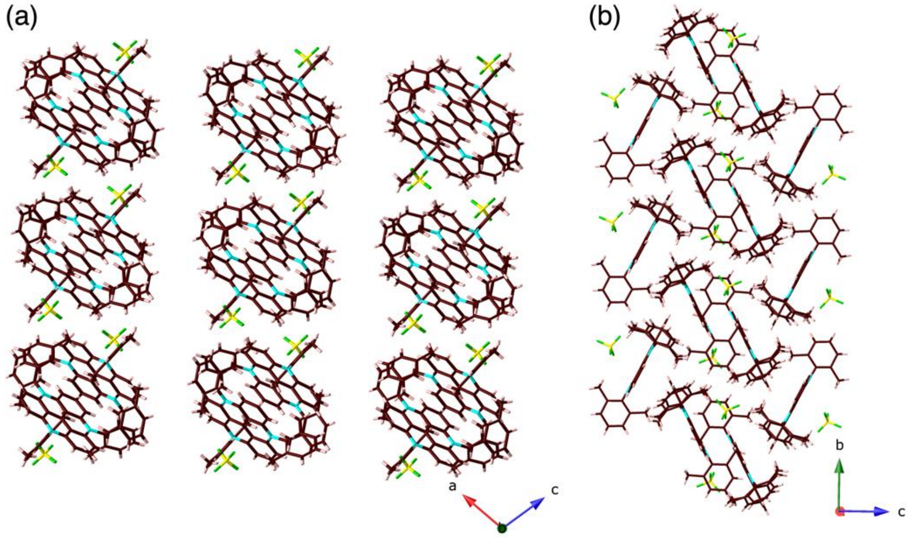

Figure S12 Single-crystal X-ray structure of **2**$^{+}$-BF_4^{-}: (a) top and (b) side views. Solvent molecules are omitted for clarity. The dihedral angle between the TATA^{+} planes was 53.7°. Atom color code: brown, pink, yellow, blue, and green refer to carbon, hydrogen, boron, nitrogen, and fluorine, respectively.

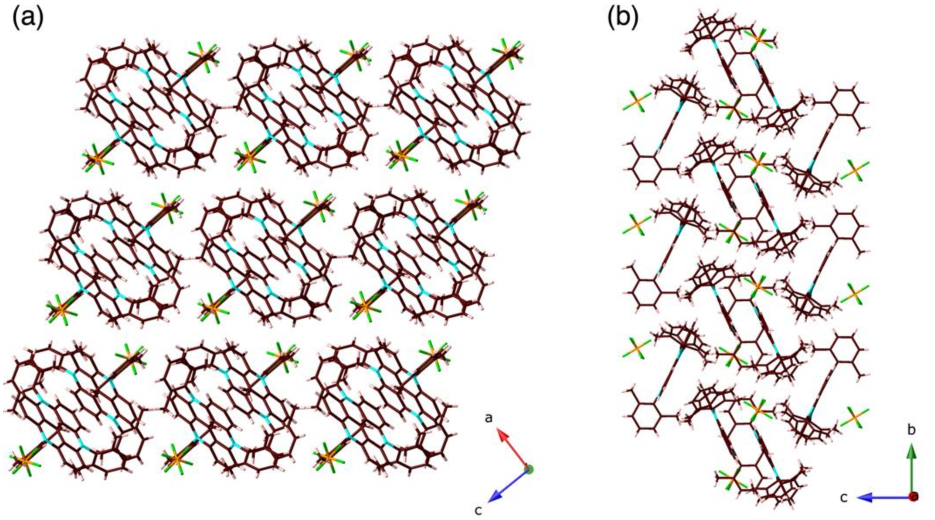

Figure S13 Single-crystal X-ray structure of **2**$^{+}$-PF_6^{-}: (a) top and (b) side views. The dihedral angle between the TATA^{+} planes was 51.4°. Atom color code: brown, pink, blue, green, and orange refer to carbon, hydrogen, nitrogen, fluorine, and phosphorus, respectively.

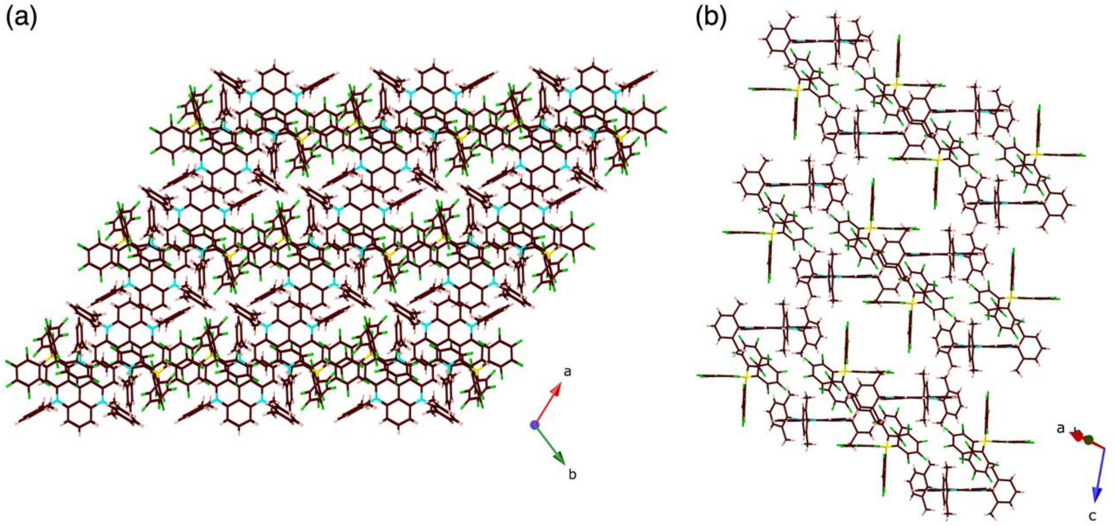

Figure S14 Single-crystal X-ray structure of **2**$^+$-$B(C_6F_5)_4^-$: (a) top and (b) side views. Solvent molecules are omitted for clarity. Atom color code: brown, pink, yellow, blue, and green refer to carbon, hydrogen, boron, nitrogen, and fluorine, respectively.

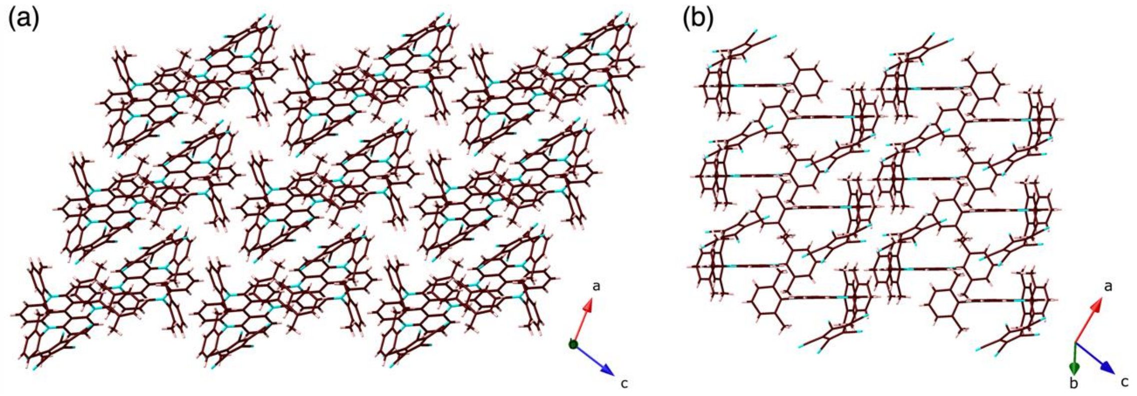

Figure S15 Single-crystal X-ray structure of **2**$^+$-PCCp$^-$: (a) top and (b) side views. Solvent molecules are omitted for clarity. The distance between the TATA$^+$ planes on the same column was 8.93 Å. Atom color code: brown, pink, and blue refer to carbon, hydrogen, and nitrogen, respectively.

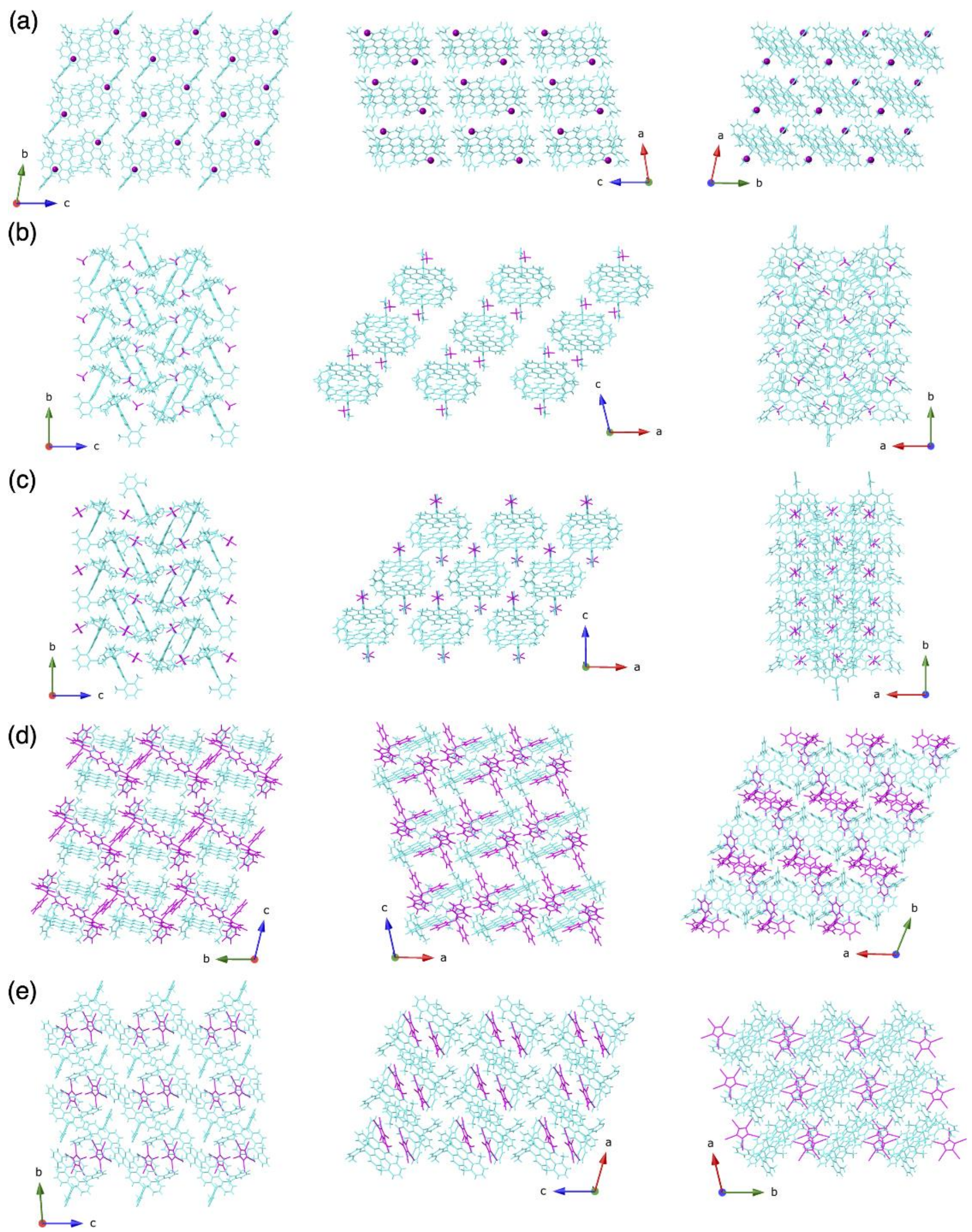

Figure S16 Packing diagrams through *a*, *b*, and *c* axes of (a) **2**$^{+}$-Cl^{-}, (b) **2**$^{+}$-BF$_4$$^{-}$, (c) **2**$^{+}$-PF$_6$$^{-}$, (d) **2**$^{+}$-B(C$_6F_5$)$_4$$^{-}$, and (e) **2**$^{+}$-PCCp^{-}, wherein cyan and magenta represent cation and anion parts, respectively.

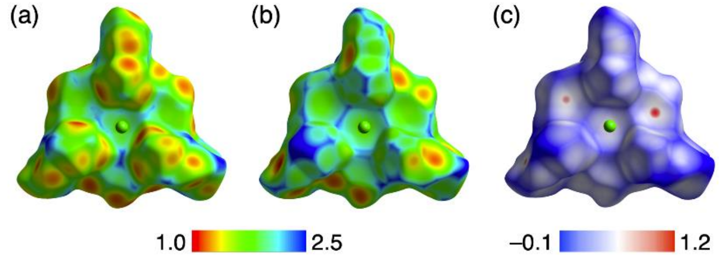

Figure S17 Hirshfeld surfaces[S2] of **2**$^+$-Cl$^-$ in the crystal structure mapped with (a) d_i, (b) d_e, and (c) d_{norm}. d_i is a distance from the Hirshfeld surface to the nearest atom inside the surface, d_e is a distance from the Hirshfeld surface to the nearest atom outside the surface, and d_{norm} is a normalized contact distance. Atom color code: green refers to chlorine.

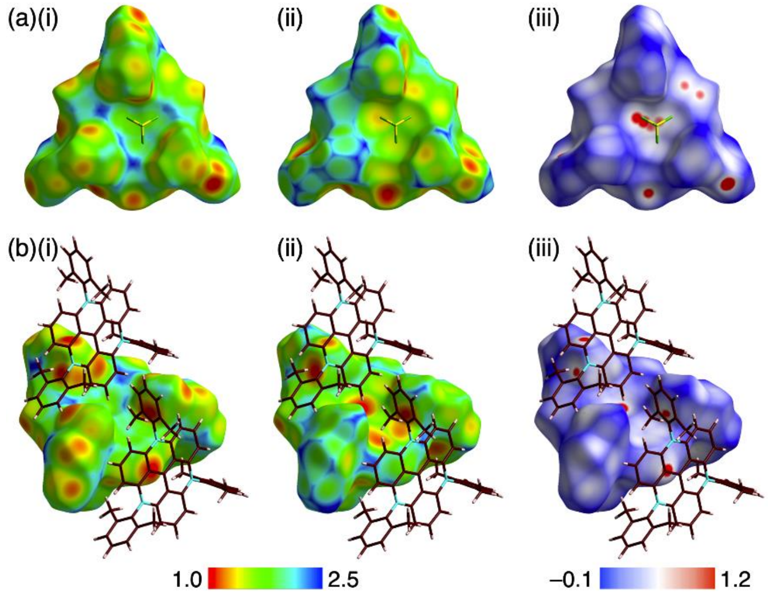

Figure S18 Hirshfeld surfaces[S2] of **2**$^+$-BF$_4^-$ in the crystal structure as (a) ion pair and (b) cations mapped with (i) d_i, (ii) d_e, and (iii) d_{norm}. d_i is a distance from the Hirshfeld surface to the nearest atom inside the surface, d_e is a distance from the Hirshfeld surface to the nearest atom outside the surface, and d_{norm} is a normalized contact distance. Atom color code: brown, pink, yellow, blue, and green refer to carbon, hydrogen, boron, nitrogen, and fluorine, respectively.

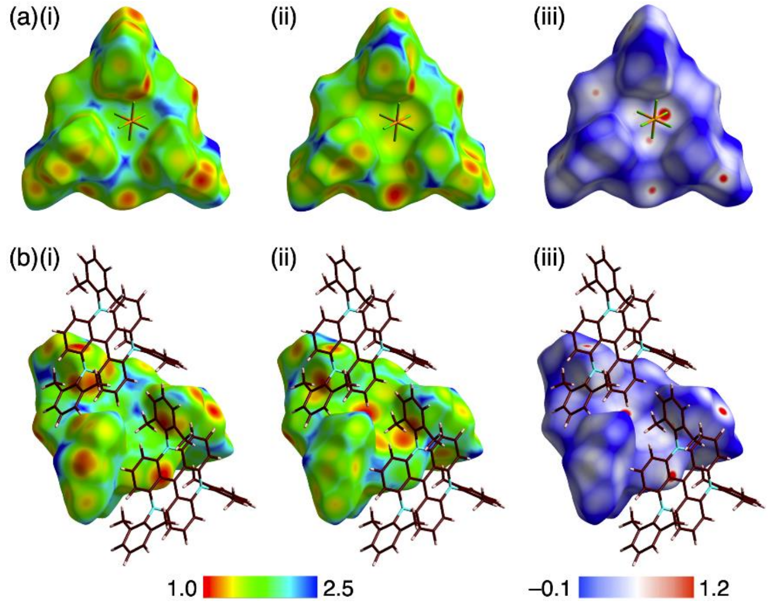

Figure S19 Hirshfeld surfaces[S2] of **2**$^+$-PF$_6^-$ in the crystal structure as (a) ion pair and (b) cations mapped with (i) d_i, (ii) d_e, and (iii) d_{norm}. d_i is a distance from the Hirshfeld surface to the nearest atom inside the surface, d_e is a distance from the Hirshfeld surface to the nearest atom outside the surface, and d_{norm} is a normalized contact distance. Atom color code: brown, pink, blue, green, and orange refer to carbon, hydrogen, nitrogen, fluorine, and phosphorus, respectively.

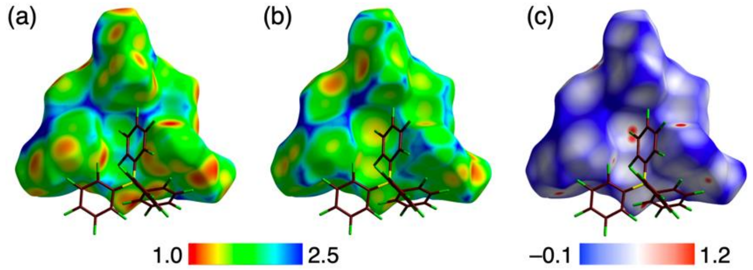

Figure S20 Hirshfeld surfaces[S2] of **2**$^+$-B(C_6F_5)$_4^-$ in the crystal structure mapped with (a) d_i, (b) d_e, and (c) d_{norm}. d_i is a distance from the Hirshfeld surface to the nearest atom inside the surface, d_e is a distance from the Hirshfeld surface to the nearest atom outside the surface, and d_{norm} is a normalized contact distance. Atom color code: brown, yellow, and green refer to carbon, boron, and fluorine, respectively.

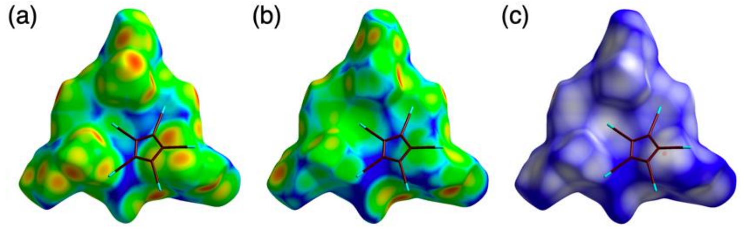

Figure S21 Hirshfeld surfaces[S2] of **2**$^+$-PCCp$^-$ in the crystal structure mapped with (a) d_i, (b) d_e, and (c) d_{norm}. d_i is a distance from the Hirshfeld surface to the nearest atom inside the surface, d_e is a distance from the Hirshfeld surface to the nearest atom outside the surface, and d_{norm} is a normalized contact distance. Atom color code: brown and blue refer to carbon and nitrogen, respectively.

[S2] Spackman, P. R.; Turner, M. J.; McKinnon, J. J.; Wolff, S. K.; Grimwood, D. J.; Jayatilaka, D.; Spackman, M. A. *J. Appl. Cryst.* **2021**, *54*, 1006–1011.

3. Theoretical studies

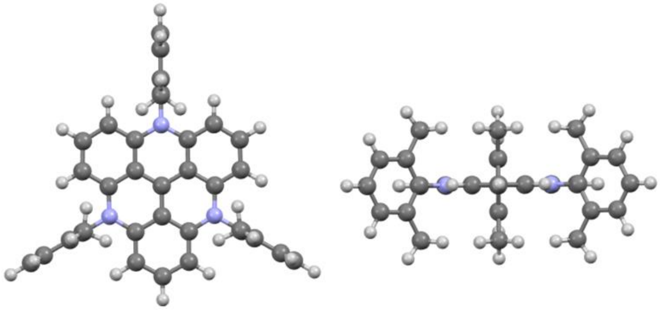

Figure S22 Optimized structure (top and side views) of **2**$^{+}$ at B3LYP/6-31+G(d,p).

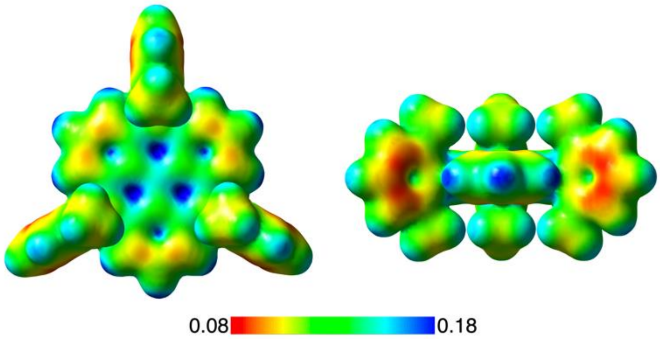

Figure S23 Electrostatic potentials (ESP) mapped onto the electron density isosurfaces (δ = 0.01) for top and side views of **2**$^{+}$ at B3LYP/6-31+G(d,p).

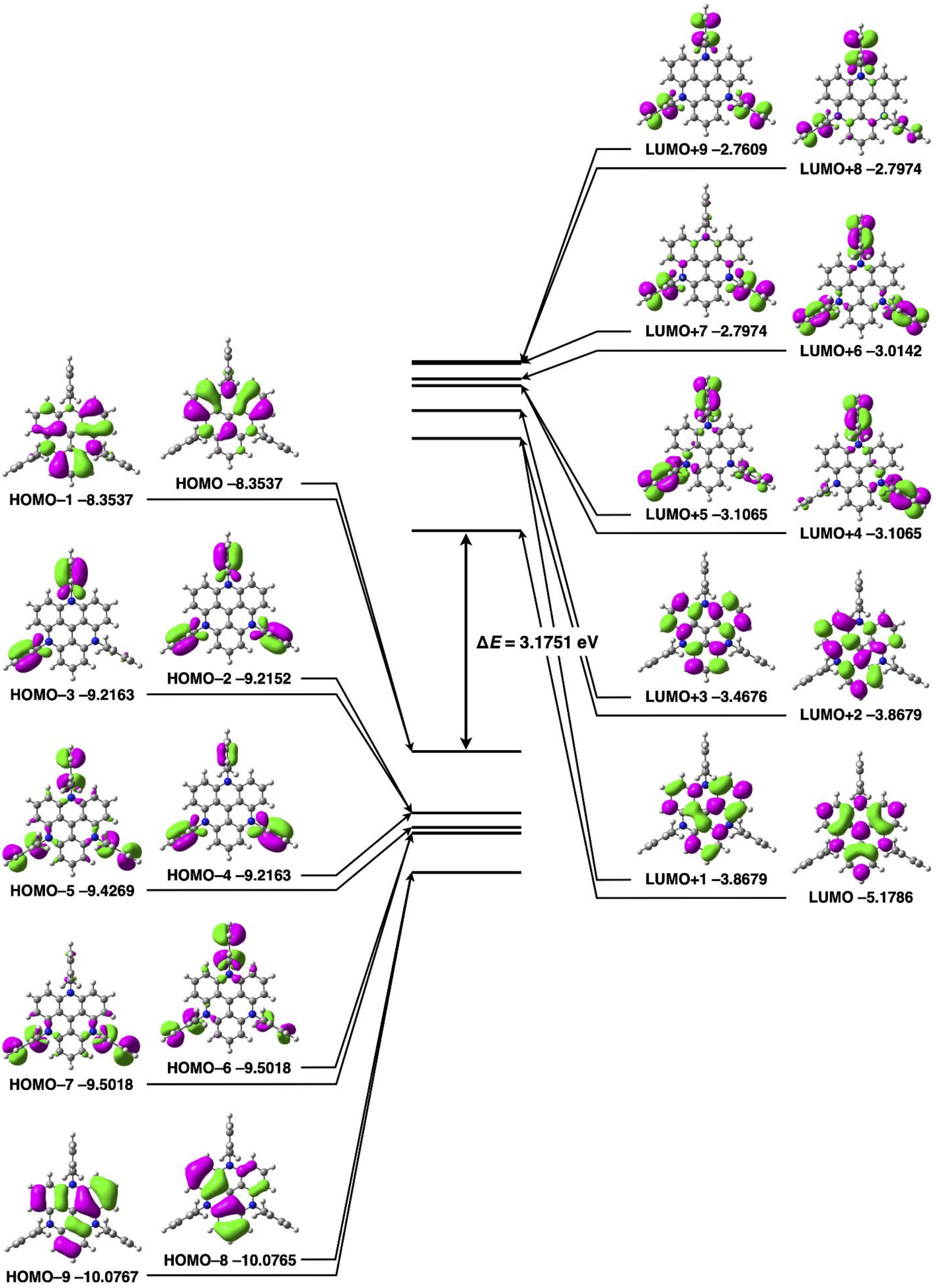

Figure S24 Molecular orbitals (HOMO/LUMO) of **2**$^{+}$ estimated at B3LYP/6-31G(d,p).

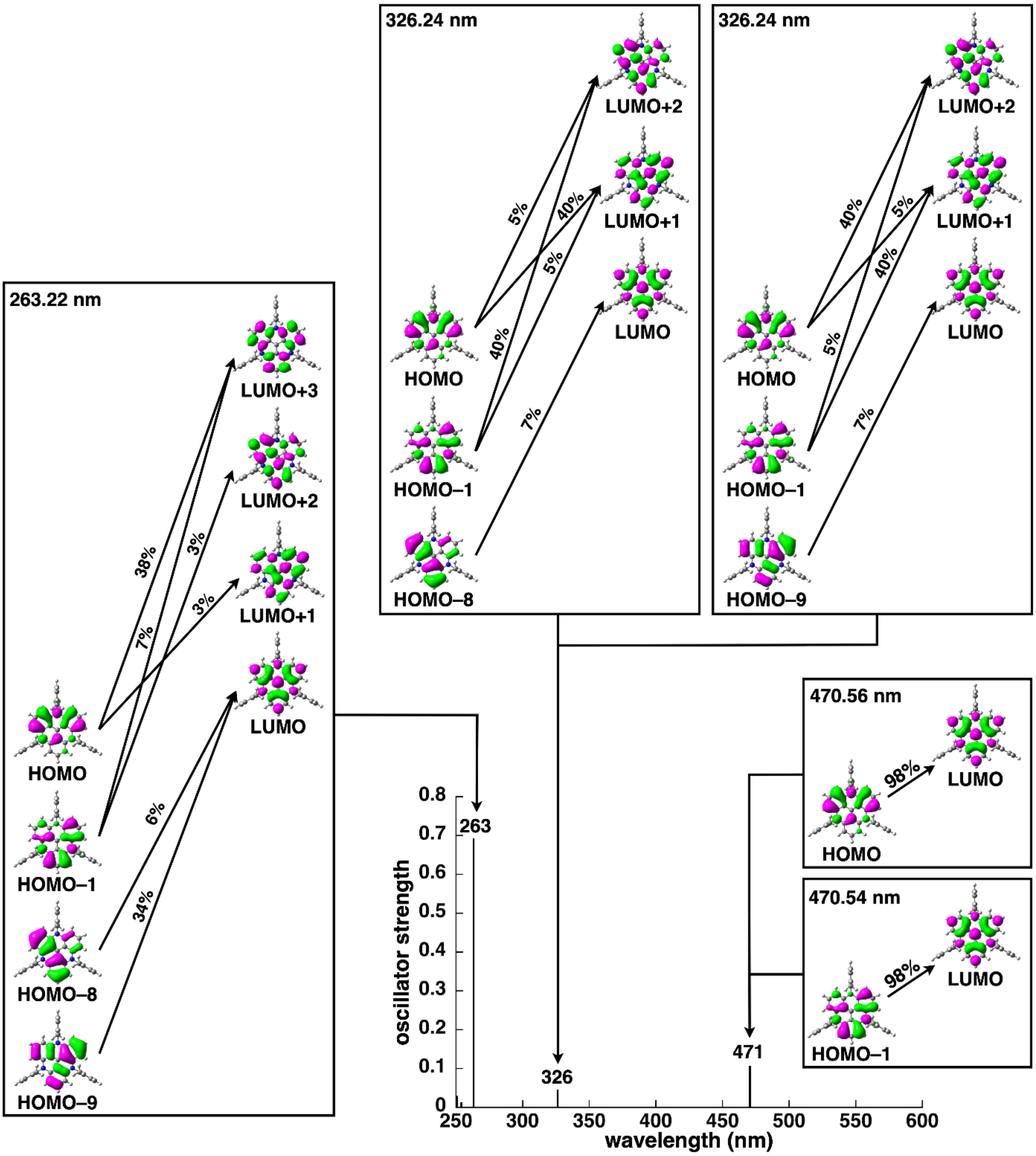

Figure S25 TD-DFT-based UV–vis absorption stick spectrum of **2**$^+$ estimated at B3LYP/6-31G(d,p).

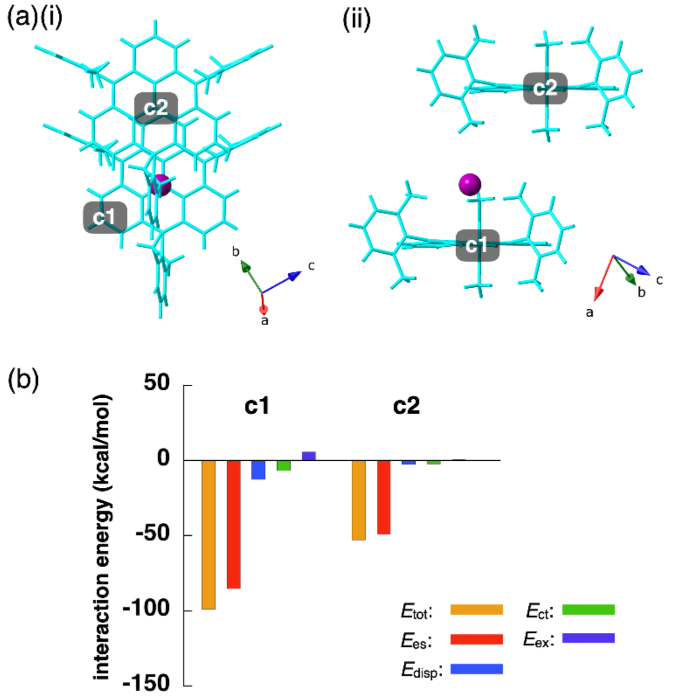

Figure S26 Single-crystal X-ray structure of **2**$^+$-Cl^- for the EDA calculations (Table S1): (a) packing structures ((i) top and (ii) side views) and (b) intermolecular interaction energies (kcal/mol) between Cl^- and selected **2**$^+$ estimated at the FMO2-MP2 method using NOSeC-V-DZP with MCP.[S3–5]

Table S1 Energies between selected fragments in **2**$^+$-Cl^- (Figure S26) estimated by EDA calculations based on an FMO2-MP2 using the basis set of NOSeC-V-DZP with MCP.[S3–5]

fragments	total interaction energy (E_{tot}) (kcal/mol)	electrostatic interaction energy (E_{es}) (kcal/mol)	dispersion interaction energy (E_{disp}) (kcal/mol)	charge-transfer interaction energy (E_{ct+mix}) (kcal/mol)	exchange repulsion interaction energy (E_{ex}) (kcal/mol)
Cl^--c1	−98.717	−85.178	–12.516	–6.653	5.540
Cl^--c2	–52.788	–49.000	–2.3600	–2.259	0.832

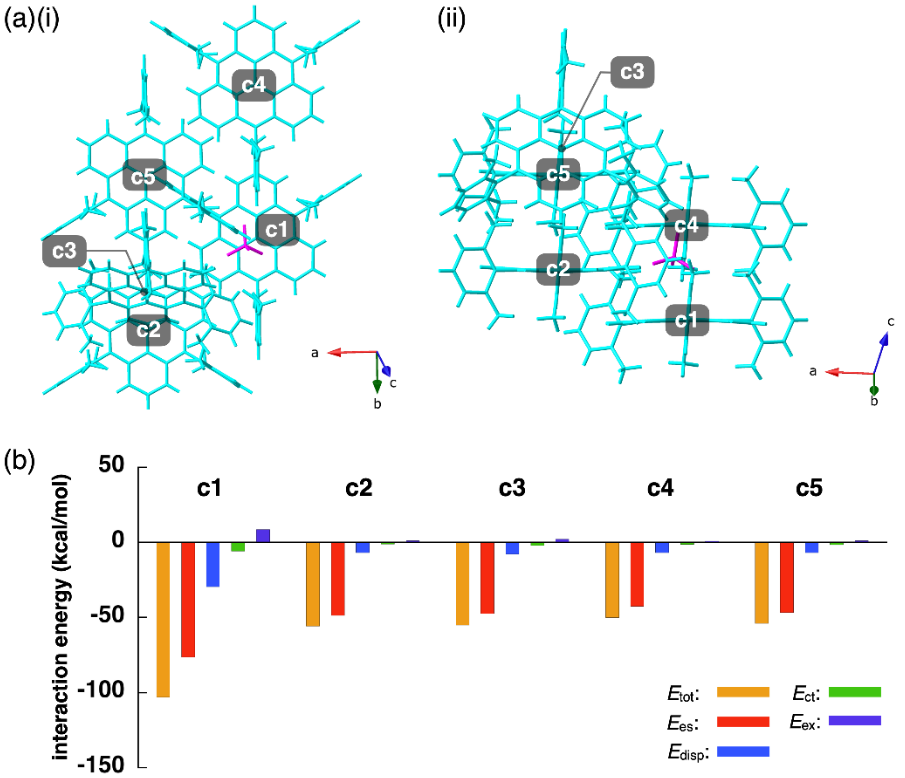

Figure S27 Single-crystal X-ray structure of **2**$^{+}$-BF_4^{-} for the EDA calculations (Table S2): (a) packing structures ((i) top and (ii) side views) and (b) intermolecular interaction energies (kcal/mol) between BF_4^{-} and selected **2**$^{+}$ estimated at the FMO2-MP2 method using NOSeC-V-DZP with MCP.[S3–5]

Table S2 Energies between selected fragments in **2**$^{+}$-BF_4^{-} (Figure S27) estimated by EDA calculations based on an FMO2-MP2 using the basis set of NOSeC-V-DZP with MCP.[S3–5]

fragments	total interaction energy (E_{tot}) (kcal/mol)	electrostatic interaction energy (E_{es}) (kcal/mol)	dispersion interaction energy (E_{disp}) (kcal/mol)	charge-transfer interaction energy (E_{ct+mix}) (kcal/mol)	exchange repulsion interaction energy (E_{ex}) (kcal/mol)
BF_4^{-}-c1	–102.928	–76.282	–29.476	–5.649	8.480
BF_4^{-}-c2	–55.712	–48.578	–6.877	–1.193	0.935
BF_4^{-}-c3	–53.964	–46.713	–6.808	–1.480	1.041
BF_4^{-}-c4	–49.887	–42.543	–6.886	–1.276	0.818
BF_4^{-}-c5	–54.956	–47.349	–7.743	–2.209	2.165

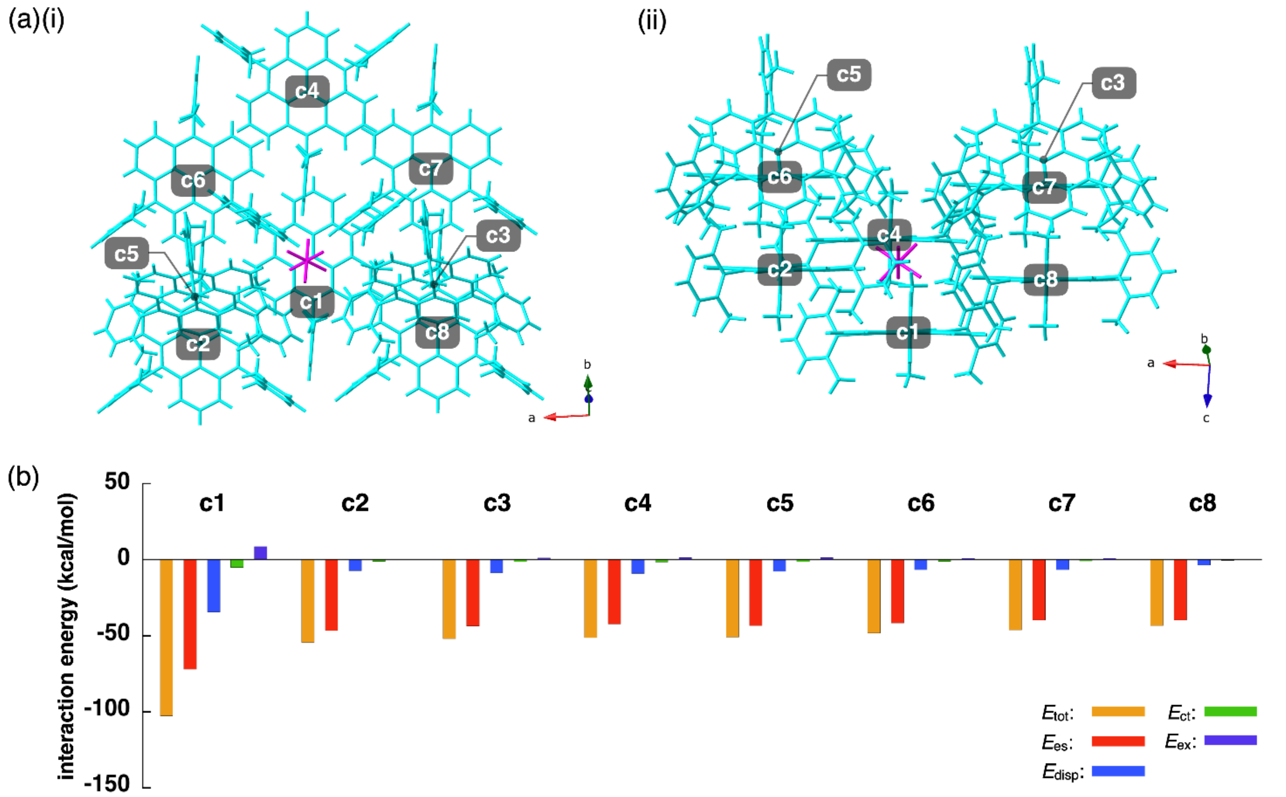

Figure S28 Single-crystal X-ray structure of **2**$^+$-PF_6^- for the EDA calculations (Table S3): (a) packing structures ((i) top and (ii) side views) and (b) intermolecular interaction energies (kcal/mol) between PF_6^- and selected **2**$^+$ estimated at the FMO2-MP2 method using NOSeC-V-DZP with MCP.[S3–5]

Table S3 Energies between selected fragments in **2**$^+$-PF_6^- (Figure S28) estimated by EDA calculations based on an FMO2-MP2 using the basis set of NOSeC-V-DZP with MCP.[S3–5]

fragments	total interaction energy (E_{tot}) (kcal/mol)	electrostatic interaction energy (E_{es}) (kcal/mol)	dispersion interaction energy (E_{disp}) (kcal/mol)	charge-transfer interaction energy (E_{ct+mix}) (kcal/mol)	exchange repulsion interaction energy (E_{ex}) (kcal/mol)
PF_6^--c1	–102.489	–71.920	–34.234	–5.012	8.677
PF_6^--c2	–54.268	–46.632	–7.228	–0.955	0.547
PF_6^--c3	–51.968	–43.416	–8.455	–1.285	1.187
PF_6^--c4	–51.127	–42.181	–8.916	–1.402	1.372
PF_6^--c5	–50.816	–43.287	–7.578	–1.292	1.341
PF_6^--c6	–48.140	–41.555	–6.562	–1.018	0.995
PF_6^--c7	–46.091	–39.493	–6.551	–0.901	0.853
PF_6^--c8	–43.390	–39.541	–3.505	–0.404	0.060

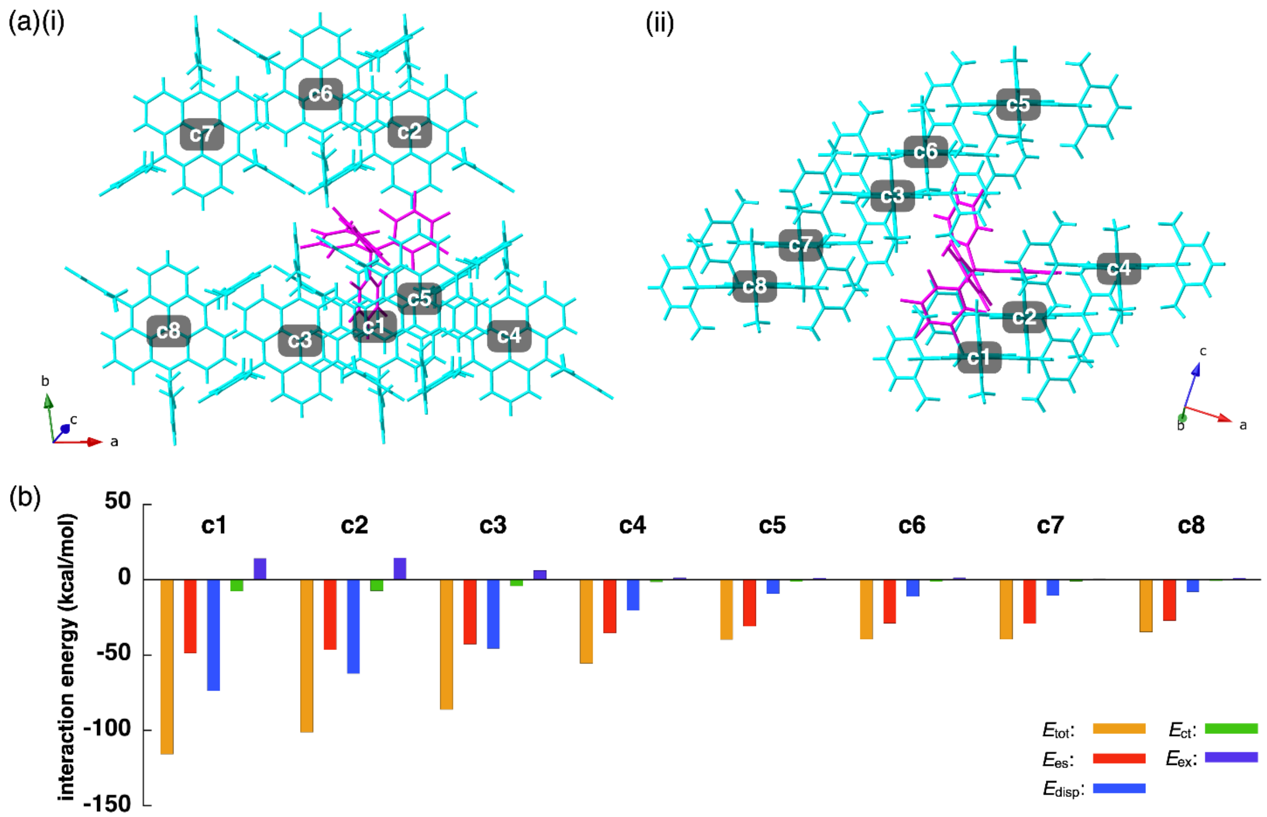

Figure S29 Single-crystal X-ray structure of **2**$^+$-B(C_6F_5)$_4^-$ for the EDA calculations (Table S4): (a) packing structures ((i) top and (ii) side views) and (b) intermolecular interaction energies (kcal/mol) between B(C_6F_5)$_4^-$ and selected **2**$^+$ estimated at the FMO2-MP2 method using NOSeC-V-DZP with MCP.[S3–5]

Table S4 Energies between selected fragments in **2**$^+$-B(C_6F_5)$_4^-$ (Figure S29) estimated by EDA calculations based on an FMO2-MP2 using basis set of NOSeC-V-DZP with MCP.[S3–5]

fragments	total interaction energy (E_{tot}) (kcal/mol)	electrostatic interaction energy (E_{es}) (kcal/mol)	dispersion interaction energy (E_{disp}) (kcal/mol)	charge-transfer interaction energy (E_{ct+mix}) (kcal/mol)	exchange repulsion interaction energy (E_{ex}) (kcal/mol)
B(C_6F_5)$_4^-$-c1	–115.698	–48.551	–73.682	–7.517	14.052
B(C_6F_5)$_4^-$-c2	–101.241	–46.392	–62.150	–7.259	14.560
B(C_6F_5)$_4^-$-c3	–86.012	–42.719	–45.520	–4.067	6.935
B(C_6F_5)$_4^-$-c4	–55.396	–35.217	–20.276	–1.282	1.379
B(C_6F_5)$_4^-$-c5	–39.621	–30.683	–9.194	–0.945	1.210
B(C_6F_5)$_4^-$-c6	–39.425	–28.828	–10.850	–1.103	1.357
B(C_6F_5)$_4^-$-c7	–39.302	–28.890	–10.229	–0.835	0.652
B(C_6F_5)$_4^-$-c8	–34.495	–27.114	–7.905	–0.676	1.199

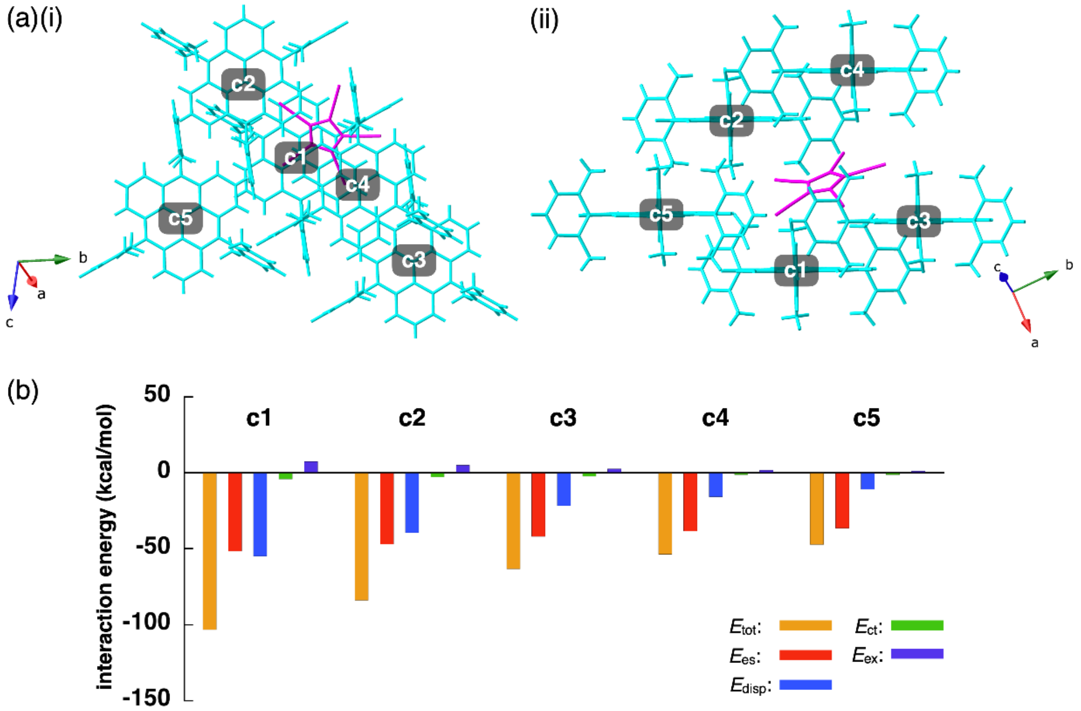

Figure S30 Single-crystal X-ray structure of **2**$^{+}$-PCCp^{-} for the EDA calculations (Table S5): (a) packing structures ((i) top and (ii) side views) and (b) intermolecular interaction energies (kcal/mol) between PCCp^{-} and selected **2**$^{+}$ estimated at the FMO2-MP2 method using NOSeC-V-DZP with MCP.[S3–5]

Table S5 Energies between selected fragments in **2**$^{+}$-PCCp^{-} (Figure S30) estimated by EDA calculations based on an FMO2-MP2 using the basis set of NOSeC-V-DZP with MCP.[S3–5]

fragments	total interaction energy (E_{tot}) (kcal/mol)	electrostatic interaction energy (E_{es}) (kcal/mol)	dispersion interaction energy (E_{disp}) (kcal/mol)	charge-transfer interaction energy (E_{ct+mix}) (kcal/mol)	exchange repulsion interaction energy (E_{ex}) (kcal/mol)
PCCp^{-}-c1	–103.180	–51.513	–54.901	–4.109	7.344
PCCp^{-}-c2	–83.820	–46.847	–39.318	–2.683	5.028
PCCp^{-}-c3	–63.111	–41.779	–21.707	–2.214	2.589
PCCp^{-}-c4	–53.593	–38.366	–15.768	–1.126	1.667
PCCp^{-}-c5	–46.988	–36.286	–10.655	–1.137	1.090

Cartesian Coordination of 2^+

–1824.5377133 hartree

C,-0.3280394164,-0.2081893775,0.6435716534
C,-1.2469212786,0.5787239715,1.3751901435
C,-2.2069791359,-0.0535496247,2.2220161073
C,-1.2101006892,2.0016521517,1.2634834181
C,-3.1115041463,0.737894834,2.941329104
C,-2.1261063566,2.7694192096,1.9938471736
C,-3.0523887458,2.1250015044,2.8128618621
H,-3.8475951236,0.2807882917,3.5890367782
H,-2.1167740751,3.8489780644,1.9248355989
H,-3.7580028886,2.729320035,3.3747551758
C,-0.3613116145,-1.617475456,0.7515256347
C,-1.3244292482,-2.2422341587,1.6004652429
C,0.5683632174,-2.4086825025,0.0110668496
C,-1.3464868964,-3.639345043,1.6982462171
C,0.5245096705,-3.8038736505,0.1271718046
C,-0.4266918242,-4.3866340294,0.9636492971
H,-2.0672787743,-4.1344659162,2.3351284151
H,1.2190022258,-4.4234033599,-0.424334791
H,-0.4522459178,-5.4688949193,1.0465580742
C,0.6241316708,0.4141770056,-0.1960076247
C,0.6545070561,1.8376829402,-0.3023502105
C,1.5504165633,-0.3840054494,-0.9332273015
C,1.6029284464,2.4414509916,-1.1377816219
C,2.4885234653,0.245367819,-1.7613811967
C,2.4950148621,1.6370919491,-1.8457830311
H,1.6445949703,3.518164098,-1.2340412871
H,3.2000439343,-0.3390356414,-2.3293064961
H,3.2261988455,2.1150424728,-2.4905685629
N,-0.2620185643,2.5884996428,0.4293243706
N,1.4955133755,-1.7698027937,-0.8082897859
N,-2.2176172842,-1.4432671994,2.3096857137
C,-1.0085688638,4.6542734005,-0.6757957358
C,-0.22791574,4.0378983413,0.3182751733
C,0.5841311454,4.7612795203,1.2097986514
C,0.6005020176,6.1566113653,1.0835107383
C,-0.1632329592,6.7965791559,0.107087402
C,-0.9594573127,6.0518051935,-0.7633100905
H,1.2185101189,6.7414077,1.758443492
H,-0.1378590906,7.8788600345,0.0242351259
H,-1.5519773456,6.5552715656,-1.5215296193
C,-5.0613070607,-3.3009404025,4.8165461577
C,-3.8134072174,-2.9432475498,5.3274742521
C,-2.8533297897,-2.3258171824,4.5149448995
C,-3.196997323,-2.083328139,3.1730787556
C,-4.4460155139,-2.4323253216,2.6293260567
C,-5.3733490406,-3.0475527156,3.4806296632
H,-5.792707515,-3.7786223239,5.4613034923
H,-3.5758320412,-3.1429953338,6.3682033116
H,-6.3463129961,-3.3282408241,3.0881842064
C,2.0811365634,-3.0065579953,-2.8512226256
C,3.0081474868,-3.7850342534,-3.5567726884
C,4.2395681987,-4.1192748504,-2.9927325412
C,4.5679484039,-3.6804998267,-1.709806949
C,3.6736787979,-2.8998283006,-0.9654892183
C,2.4406074733,-2.5791487106,-1.560639434
H,2.7577325959,-4.1294351361,-4.5558405475
H,4.9453249355,-4.7235087071,-3.5545595466
H,5.5279520646,-3.9437886696,-1.2756084391
C,1.411077798,4.0687950647,2.2672072895
H,2.1331431379,3.373621747,1.8244840163
H,1.9699583796,4.7992879092,2.8561330784
H,0.7844117951,3.4924728617,2.9569977367
C,-1.8709294306,3.8483004924,-1.6182536998
H,-1.2746910403,3.1442360956,-2.2093801176
H,-2.6236964388,3.2640222788,-1.0772913698
H,-2.396505215,4.5058676641,-2.3140817327
C,-4.7843127183,-2.1582320042,1.1829786772
H,-5.7956285282,-2.5012845591,0.9541601298
H,-4.7341018459,-1.0882842258,0.952013838
H,-4.0954292653,-2.6713060599,0.5026477755
C,-1.5023157758,-1.9388135055,5.0685560955
H,-0.6880552396,-2.4436265895,4.5367635714
H,-1.3259262736,-0.8604607061,4.9867527918
H,-1.4288057734,-2.2092241039,6.1241490971
C,4.0295210811,-2.4252450224,0.4236252056
H,4.0298625771,-1.3314757608,0.4892756391
H,3.3199839309,-2.7965191199,1.1715462902
H,5.0246180512,-2.7761864396,0.7054482145
C,0.7478767558,-2.6451483164,-3.4622011187
H,-0.0873235719,-3.0248968988,-2.863114689
H,0.6224021598,-1.5598057218,-3.5454398553
H,0.6582468648,-3.0687164339,-4.4648768616

[S3] Articles for *GAMESS*: (a) Schmidt, M. W.; Baldridge, K. K.; Boatz, J. A.; Elbert, S. T.; Gordon, M. S.; Jensen, J. H.; Koseki, S.; Matsunaga, N.; Nguyen, K. A.; Su, S.; Windus, T. L.; Dupuis, M.; Montgomery, J. A. *J. Comput. Chem.* **1993**, *14*, 1347–1363. (b) Gordon, M. S.; Schmidt, M. W. In *Theory and Applications of Computational Chemistry: the first forty years*; Dykstra, C. E., Frenking, G., Kim, K. S., Scuseria, G. E. Eds.; Elsevier, 2005, pp. 1167–1189.

[S4] Report for FMO: Kitaura, K.; Ikeo, E.; Asada, T.; Nakano, T.; Uebayasi, M. *Chem. Phys. Lett.* **1999**, *313*, 701–706.

[S5] Report for pair interaction energy decomposition analysis (PIEDA): Fedorov, D. G.; Kitaura, K. *J. Comput. Chem.* **2007**, *28*, 222–237.
